# AI-Guided Inference of Morphodynamic Attractor-like States in Glioblastoma

**DOI:** 10.3390/diagnostics16010139

**Published:** 2026-01-01

**Authors:** Simona Ruxandra Volovăț, Diana Ioana Panaite, Mădălina Raluca Ostafe, Călin Gheorghe Buzea, Dragoș Teodor Iancu, Maricel Agop, Lăcrămioara Ochiuz, Dragoș Ioan Rusu, Cristian Constantin Volovăț

**Affiliations:** 1 Department of Oncology-Radiotherapy, University of Medicine and Pharmacy “Grigore T. Popa” Iași, 700115 Iași, Romania; simonavolovat@gmail.com (S.R.V.); dianaiboboc@gmail.com (D.I.P.); madalina.ostafe@gmail.com (M.R.O.); 2 National Institute of Research and Development for Technical Physics, IFT Iași, 700050 Iași, Romania; calinb2003@yahoo.com; 3 Clinical Emergency Hospital “Prof. Dr. Nicolae Oblu” Iași, 700309 Iași, Romania; 4 Regional Institute of Oncology, IRO Iași, 700483 Iași, Romania; 5 Faculty of Medicine, University of Medicine and Pharmacy “Grigore T. Popa” Iași, 700115 Iași, Romania; ochiuzd@yahoo.com; 6 Department of Environmental Engineering, Mechanical Engineering and Agritourism, Faculty of Engineering, “Vasile Alecsandri” University of Bacău, 600115 Bacău, Romania; m.agop@yahoo.com (M.A.); drusu@ub.ro (D.I.R.); 7 Department of Radiology, University of Medicine and Pharmacy “Grigore T. Popa” Iași, 700115 Iași, Romania; cristian.volovat@yahoo.com

**Keywords:** glioblastoma, latent dynamics, morphodynamic attractors, deep learning, survival analysis, radiomics, nonlinear dynamical systems, tumor evolution, clinical stratification

## Abstract

**Background/Objectives**: Glioblastoma (GBM) exhibits heterogeneous, nonlinear invasion patterns that challenge conventional modeling and radiomic prediction. Most deep learning approaches describe the morphology but rarely capture the dynamical stability of tumor evolution. We propose an AI framework that approximates a latent attractor landscape of GBM morphodynamics—stable basins in a continuous manifold that are consistent with reproducible morphologic regimes. **Methods**: Multimodal MRI scans from BraTS 2020 (n = 494) were standardized and embedded with a 3D autoencoder to obtain 128-D latent representations. Unsupervised clustering identified latent basins (“attractors”). A neural ordinary differential equation (neural-ODE) approximated latent dynamics. All dynamics were inferred from cross-sectional population variability rather than longitudinal follow-up, serving as a proof-of-concept approximation of morphologic continuity. Voxel-level perturbation quantified local morphodynamic sensitivity, and proof-of-concept control was explored by adding small inputs to the neural-ODE using both a deterministic controller and a reinforcement learning agent based on soft actor–critic (SAC). Survival analyses (Kaplan–Meier, log-rank, ridge-regularized Cox) assessed associations with outcomes. **Results**: The learned latent manifold was smooth and clinically organized. Three dominant attractor basins were identified with significant survival stratification (χ^2^ = 31.8, *p* = 1.3 × 10^−7^) in the static model. Dynamic attractor basins derived from neural-ODE endpoints showed modest and non-significant survival differences, confirming that these dynamic labels primarily encode the morphodynamic structure rather than fixed prognostic strata. Dynamic basins inferred from neural-ODE flows were not independently prognostic, indicating that the inferred morphodynamic field captures geometric organization rather than additional clinical risk information. The latent stability index showed a weak but borderline significant negative association with survival (ρ = −0.13 [−0.26, −0.01]; *p* = 0.0499). In multivariable Cox models, age remained the dominant covariate (HR = 1.30 [1.16–1.45]; *p* = 5 × 10^−6^), with overall C-indices of 0.61–0.64. Voxel-level sensitivity maps highlighted enhancing rims and peri-necrotic interfaces as influential regions. In simulation, deterministic control redirected trajectories toward lower-risk basins (≈57% success; ≈96% terminal distance reduction), while a soft actor–critic (SAC) agent produced smoother trajectories and modest additional reductions in terminal distance, albeit without matching the deterministic controller’s success rate. The learned attractor classes were internally consistent and clinically distinct. **Conclusions**: Learning a latent attractor landscape links generative AI, dynamical systems theory, and clinical outcomes in GBM. Although limited by the cross-sectional nature of BraTS and modest prognostic gains beyond age, these results provide a mechanistic, controllable framework for tumor morphology in which inferred dynamic attractor-like flows describe latent organization rather than a clinically predictive temporal model, motivating prospective radiogenomic validation and adaptive therapy studies.

## 1. Introduction

### 1.1. Glioblastoma and the Challenge of Predicting Its Evolution

Glioblastoma (GBM) is the most aggressive and treatment-resistant primary brain tumor in adults, characterized by rapid infiltrative growth, cellular heterogeneity, and inevitable recurrence despite maximal therapy. Prognosis remains dismal, with a median survival duration of approximately 15 months under the current standard-of-care regimen combining maximal resection, radiotherapy, and temozolomide chemotherapy [1,2].

A central challenge in GBM research lies in understanding and anticipating the spatiotemporal dynamics of tumor progression, which are highly nonlinear and patient-specific, driven by feedback between cellular proliferation, microenvironmental pressures, and treatment-induced stress. In this work, we use the term ‘morphodynamics’ to denote the inferred patterns of morphologic change implied by population-level variability on a latent manifold, rather than directly observed longitudinal evolution. Capturing these dynamics from imaging remains difficult, as standard radiomic pipelines quantify the morphology but not its evolution over time.

Over the last decade, the neuroimaging community has embraced radiomics and deep learning approaches to quantify GBM’s morphology and heterogeneity from MRI [3,4,5,6,7,8]. However, most of these frameworks remain descriptive—they map imaging phenotypes to outcomes without capturing how the tumor state evolves or transitions between stable configurations. In other words, they lack a causal and dynamical interpretation of the disease process.

### 1.2. Limitations of Current Generative and Radiomic Approaches

Recent advances in deep generative modeling—particularly variational autoencoders (VAEs), diffusion models, and normalizing flows—have opened up new avenues for data-driven tumor phenotyping and progression synthesis [9,10,11,12,13].

VAEs and their variants have been used to learn compressed latent representations of MR volumes, enabling the clustering of patient subtypes or synthetic data generation [14,15,16]. Diffusion models have achieved remarkable realism in image synthesis [17,18,19,20], but they remain statistical samplers rather than interpretable dynamical systems.

Neither radiomics nor diffusion-based generative models explain what governs transitions between one anatomical configuration and another—or whether such transitions are stable, reversible, or sensitive to small perturbations. Consequently, despite technical sophistication, current AI-based imaging pipelines do not provide a mechanistic view of tumor morphodynamics; they characterize the appearance but not the underlying behavior in a high-dimensional morphogenetic space.

Recent studies combining deep generative models with differential equation frameworks have suggested that latent representations can encode continuous in silico biological dynamics [21,22,23], but such approaches remain largely unexplored in neuro-oncology.

To move beyond descriptive morphology, a framework is needed that models GBM as a dynamical system—capturing not only appearance but also the stability and potential transitions between tumor states. This motivates our attractor-based approach.

### 1.3. The Concept of Attractor States in Nonlinear Systems and Biological Networks

In theoretical biology and nonlinear systems science, attractors represent stable configurations of a dynamic system toward which trajectories converge over time [24,25]. Attractors can correspond to steady states, oscillations, or chaotic regimes; in gene-regulatory and metabolic networks, they have been used to formalize cell fate decisions—for example, transitions between proliferative, invasive, or quiescent phenotypes [19,26,27,28].

Recent work has directly explored glioblastoma as a nonlinear dynamical system using generative AI and attractor-based modeling. Uthamacumaran and Craig demonstrated the algorithmic reconstruction of GBM’s network complexity using generative neural architectures, highlighting that tumor phenotypes may emerge from attractor-like basins in high-dimensional molecular landscapes [29]. More recently, Uthamacumaran applied deep learning-based feature discovery to pediatric high-grade glioma at the single-cell level, revealing phenotypic plasticity consistent with attractor transitions in a gene-regulatory space [30]. These studies reinforce the view that gliomas exhibit attractor-like behavior across biological scales—from molecular states to macroscopic morphologies—and support the extension of attractor theory to radiologic morphodynamics.

Similarly, in the context of cancer evolution, attractors have been proposed as basins of stability representing dominant phenotypic programs of the tumor ecosystem [20,31]. By analogy, GBM can be viewed as a complex adaptive system whose observable morphology is a projection of a latent, high-dimensional attractor landscape shaped by genetic, microenvironmental, and therapeutic forces.

Tumor recurrence, for instance, can be conceptually interpreted as a transition from one attractor basin to another, triggered by therapy-induced perturbations that push the system across an unstable boundary in latent space. This conceptual bridge—between dynamical systems and tumor imaging phenotypes—remains largely unexplored in neuro-oncology.

### 1.4. Hypothesis: GBM Morphodynamics Evolve on a Low-Dimensional Manifold of Stable Basins

We hypothesize that GBM evolution unfolds on a low-dimensional manifold that can be learned directly from volumetric MRI using deep generative models. Within this manifold, each point encodes a tumor configuration, and the local geometry—particularly the basins (stable regions) and ridges (unstable boundaries) of the learned latent landscape—reflects the stability of morphological patterns.

While this manifold does not represent real-time dynamics, its geometry can approximate the relative stability and potential transition pathways between morphologic states.

From this perspective,

- Stable attractor basins may correspond to recurring or treatment-resistant morphotypes;
- Steep basin walls represent resistance to small perturbations (therapeutic or biological);
- Shallow regions indicate instability or potential for morphological reprogramming.

This view transforms the problem of tumor interpretation from “which features correlate with outcomes?” to “how stable is the current morphological state, and what perturbations might induce a transition toward a more favorable attractor?”

### 1.5. Aim and Novelty of the Present Study

To explore this hypothesis, we developed a framework that combines

1. The unsupervised autoencoding of 3D MR volumes to learn a latent attractor landscape of the tumor morphology;
2. Attractor identification using unsupervised clustering in the latent space (K-means over latent embeddings);
3. Localized perturbation analysis in the input space to probe the sensitivity of each voxel to attractor transitions.

In contrast to previous interpretability approaches (e.g., gradient saliency, class activation maps, or counterfactuals), our method explicitly quantifies the local stability of the learned attractor landscape. We define attractor stability maps as voxel-wise fields describing how small, spatially localized perturbations in the input space affect the system’s attractor membership in latent space.

This provides the first mechanistically grounded, dynamical interpretation of unsupervised morphological embeddings in GBM.

Technically, the method differs from classical sensitivity analysis in three key ways:

- It operates in a fully unsupervised setting, where attractors emerge naturally from the data rather than from labeled outcomes;
- It measures state transitions (cluster flips) rather than simple feature gradients, quantifying local basin boundaries in latent space;
- It yields a spatial map of morphological control, identifying anatomical regions that govern attractor stability—a proxy for potential therapeutic leverage points.

Although the framework operates on cross-sectional data, its structure allows the inference of latent transition directions consistent with the observed morphologic variability. Building on these static analyses, we further model the temporal geometry of the latent manifold using a neural ordinary differential equation (neural-ODE) that approximates continuous morphodynamic flow. Finally, we introduce proof-of-concept control simulations—both deterministic and reinforcement learning-based—to test whether small, structured inputs can steer trajectories within this latent attractor landscape toward lower-risk basins.

We selected a neural ordinary differential equation (neural-ODE) rather than recurrent or CNN-based generative models because neural-ODEs impose a continuous-time dynamical prior on the latent space. This results in smooth vector fields, well-defined trajectories, and stable numerical integration—properties that align naturally with nonlinear systems analysis and are not guaranteed by discrete CNN transitions. Neural-ODEs also provide explicit access to dynamical quantities such as Jacobians, divergence, and Lyapunov exponents, enabling the principled estimation of latent stability and basin geometry. These characteristics make neural-ODEs a uniquely suitable architecture for studying attractor-like morphodynamic behavior in glioblastoma.

Because these dynamics are inferred from cross-sectional data, the resulting attractor-like states should be interpreted as latent morphodynamic structures rather than clinically predictive temporal trajectories.

To assess the biological and clinical plausibility of the learned morphodynamic states, we further validate the framework on the BraTS 2020 cohort by examining whether dynamic attractor labels (derived from neural-ODE endpoint basins) exhibit consistent trends in survival and morphological composition. Although the attractor indices themselves are numerically arbitrary, their distribution across cases provides an opportunity to test whether the latent basins correspond to reproducible clinical or morphologic patterns, beyond purely geometric organization.

**Figure 1:**
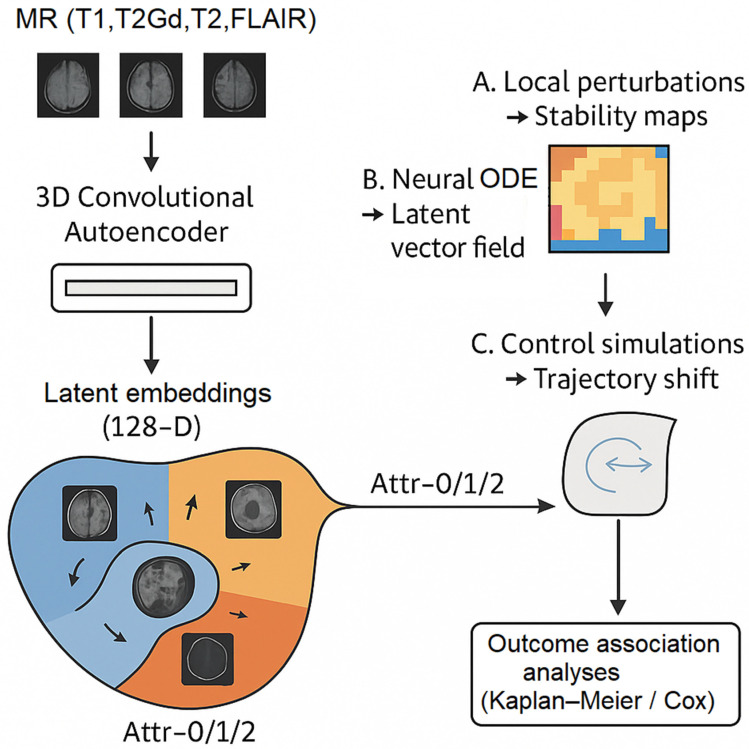
Conceptual overview of the latent attractor framework. Multimodal MR images (T1, T1Gd, T2, FLAIR) are encoded by a 3D convolutional autoencoder into a 128-dimensional latent space, where unsupervised clustering identifies three attractor basins (Attr-0/1/2). Local perturbation analysis quantifies voxel-wise stability maps, while a neural ordinary differential equation (neural-ODE) models latent dynamics, and control simulations test trajectory shifts between basins. The resulting attractor structure was evaluated through survival stratification on the BraTS 2020 cohort.

The complete computational workflow—from MRI encoding to attractor discovery, perturbation analysis, and survival association—is summarized schematically in Figure 1.

### 1.6. Illustrative Analogy and Potential Implications

Conceptually, this approach parallels energy landscape analysis in molecular dynamics or Lyapunov stability analysis in control theory, but it is applied to data-driven manifolds of medical images. Each patient’s MRI scan can be viewed as a sample from an underlying potential field of morphological configurations, where our model estimates the local curvature (stability) and potential transitions.

This formulation provides several potential advantages:

- It offers a quantitative measure of tumor stability that may complement molecular or radiomic risk factors;
- It may identify regions of morphological fragility, indicating where local therapies or dose modulation could potentially modulate attractor stability;
- It establishes a theoretical bridge linking generative AI, dynamical systems modeling, and oncologic decision support.

Recent studies in single-cell dynamics and neurodegeneration have shown that attractor-based dynamics learned by neural-ODEs can approximate real biological trajectories [32,33]. Motivated by these findings, our framework extends the same principle to tumor morphodynamics, providing a computational lens for studying GBM evolution and exploring potential control strategies in silico.

Although the present analysis focuses on the four conventional BraTS modalities (T1, T1Gd, T2, FLAIR), the framework is inherently modality-agnostic and can readily incorporate advanced neuro-oncologic sequences. Perfusion MRI (e.g., rCBV/rCBF), diffusion tensor imaging (DTI), and MR spectroscopy provide complementary vascular, microstructural, and metabolic information that may sharpen the basin geometry and enhance the biological specificity—particularly in distinguishing hypoxic, mesenchymal-like morphologies from proneural or classical states. Integrating these advanced modalities represents an important next step in expanding the mechanistic and clinical relevance of morphodynamic attractor modeling in GBM.

### 1.7. Structure of the Paper and Objectives

The remainder of this paper is organized as follows.

- Section 2—Methods: Describes the BraTS 2020 dataset, preprocessing pipeline, and 3D autoencoder architecture used to learn the latent attractor landscape. It details procedures for attractor identification, voxel-level perturbation analysis, latent dynamics modeling with neural-ODEs, and proof-of-concept control simulations, together with validation and prospective biological integration plans.
- Section 3—Results: Presents the learned latent manifold and the discovery of three stable attractor basins, followed by analyses of latent stability, survival stratification, voxel-level sensitivity maps, latent space dynamics, and simulated control experiments.
- Section 4—Discussion: Interprets the latent attractor landscape in biological and dynamical terms, compares it with known GBM ecosystems and molecular subtypes, and discusses methodological limitations, clinical relevance, and future directions for longitudinal validation.
- Section 5—Conclusions: Summarizes the conceptual and technical contributions, emphasizing how latent attractor modeling unifies generative AI, dynamical systems theory, and clinical imaging, and outlines next steps toward biologically grounded, controllable models of tumor evolution.

Finally, the overarching objective of this study is to test whether latent embeddings of GBM MRI scans form reproducible, stable clusters (“attractors”) that correlate with patient outcomes and can be modulated through simulated control inputs—thus establishing a bridge between data-driven morphological modeling and dynamical systems approaches in oncology.

## 2. Materials and Methods

### 2.1. Data

**Clinical MRI dataset**

We used the Brain Tumor Segmentation (BraTS) 2020 dataset [7,34,35] (n = 494; 369 training, 125 validation), which provides preoperative, multiparametric MRI for patients with histologically confirmed glioblastoma (GBM).

Each subject includes four co-registered MRI sequences: T1-weighted (T1), Gadolinium-enhanced T1-weighted (T1Gd), T2-weighted (T2), and fluid-attenuated inversion recovery (FLAIR).

Expert tumor segmentations are provided only for the training subset.

These manual labels delineate three subregions—enhancing tumor (ET), tumor core (TC), and whole tumor (WT)—and were created by board-certified neuroradiologists following the official BraTS annotation protocol [34,35].

The validation subset contains only the four MRI modalities, with ground truth labels withheld to enable unbiased evaluation during the challenge [7].

Demographic and clinical outcome variables (age, extent of resection, survival days, event indicator) were merged from *survival_info.csv* (training) and *survival_evaluation.csv* (validation) into a unified metadata file (*metadata_final.csv*).

The BraTS 2020 dataset does not include molecular annotations such as the IDH1/IDH2 mutation status, MGMT promoter methylation, or transcriptional subtype. As a result, molecular–genomic stratification (e.g., IDH-wildtype vs. IDH-mutant gliomas) could not be incorporated into the present analysis. This limitation is explicitly acknowledged in the Discussion and motivates future work using datasets that provide matched imaging and molecular profiles for radiogenomic integration.

All data are fully de-identified and publicly available via The Cancer Imaging Archive (TCIA); no additional IRB approval was required.

**Figure 2:**
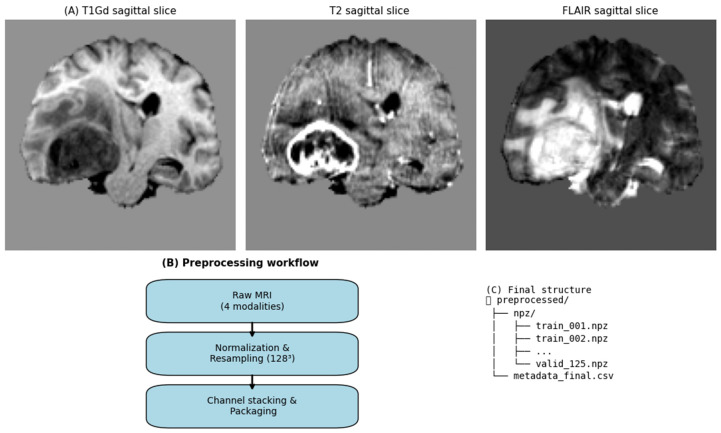
Overview of the preprocessing pipeline. (**A**) Example BraTS 2020 subject showing T1Gd, T2, and FLAIR modalities. (**B**) Preprocessing workflow: per-modality normalization, resampling to 128^3^, and channel stacking with packaging into .npz archives. (**C**) Final dataset structure with training and validation splits and metadata_final.csv. Note: During training, 64^3^ random patches were sampled from these standardized 128^3^ volumes.

A compact overview of the dataset and preprocessing workflow appears in Figure 2 and further implementation details are provided in Supplementary Materials S1.

The cohort composition and input structure are summarized in the dataset composition table (Table 1), while Table 2 provides aggregated demographic and outcome characteristics (mean age, resection categories, survival availability).

**Rationale for dataset choice**

BraTS 2020 is a large, curated public benchmark with harmonized preoperative multimodal MRI and expert-validated tumor segmentations, enabling reproducible morphometric analyses across institutions and scanner vendors.

A known limitation is its single-timepoint design, which precludes the direct observation of longitudinal tumor evolution.

Accordingly, the present work treats the dynamical systems and control components as proof-of-concept simulations to be validated prospectively when longitudinal imaging becomes available.

**Inclusion/Exclusion and Quality Control (QC)**

All official BraTS 2020 cases containing the complete four-modality MRI set and valid NIfTI headers were retained.

Automated QC (modality presence and header integrity) flagged no exclusions; therefore, the final analytic cohort comprised n = 494 subjects.

### 2.2. Preprocessing Pipeline

Environment and tooling. Python 3.10; NiBabel (I/O), TorchIO (resampling, patching, augmentation), NumPy (array ops). Processing ran locally (Windows) with synchronization to Google Drive for GPU training (Google Colab).

Steps (see Figure 2):

1. Parsing and QC. Verify presence/readability of T1, T1Gd, T2, FLAIR (NIfTI .nii.gz), record voxel size/orientation; fail closed if any header is invalid.
2. Spatial standardization. Resample each modality to 128 × 128 × 128 at 1 mm3 using trilinear interpolation (nearest-neighbor for masks). Harmonized lattice used for case-level ops and storage.
3. Per-modality intensity normalization. Clip to [1st, 99th] percentile per volume; then, linearly rescale to [0, 1] to reduce scanner bias and shrink outlier tails while preserving contrast.
4. Channel stacking. Concatenate the four normalized modalities to a 4 × 1283 tensor; train-only optional: append segmentation as channel 5 in ablation runs (never used in validation/inference).
5. Packaging. Save each case as compressed .npz (*train_XXX.npz, valid_XXX.npz*) with paths and metadata in *metadata_final.csv*.
6. Patch-based training. Random 643 3D crops drawn on-the-fly from the 1283 tensor to increase spatial diversity and fit GPU memory. Batches shaped (B, C, 64, 64, 64), with C = 4 (modalities) or C = 5 (modalities + seg; train-only ablation).

On-disk layout. /preprocessed/npz/ (case tensors) + *metadata_final.csv*.

Clarification of segmentation channel. The mask was never a prediction target; it was an auxiliary input channel in some training runs to encourage anatomic sensitivity. All downstream analyses (embeddings, clustering, survival, dynamics) used 4-channel inputs to avoid label leakage. The comprehensive code structure, command examples, and quality control metrics are documented in Supplementary Materials S1.

### 2.3. Latent Space Model (3D Autoencoder)

**Input.** A multimodal patch $x\in R^{4\times 64\times 64\times 64}$.

Encoder. Four Conv3D blocks with stride 2 and ReLU, channel progression 32 → 64 → 128 → 256, each followed by BatchNorm; global pooling + FC to a 128-D vector $z=f_{enc}(x)$.

Decoder. Mirror via ConvTranspose3D blocks with stride 2 to reconstruct $\hat{x}=f_{dec}(z)$.

**Objective.**

$$L_{AE}={\left\|x-\hat{x}\right\|}_{1}$$

Optimization and training schedule. Adam (lr = 10^−3^), batch = 2, random axis flips; early stopping on validation loss with patience = 15; max epochs = 150 (typical convergence ≤ 80). Trained in PyTorch 2.2 on an NVIDIA T4 GPU (Colab). Random seeds fixed (global = 42; PyTorch/CUDA/NumPy synchronized).

**Figure 3:**
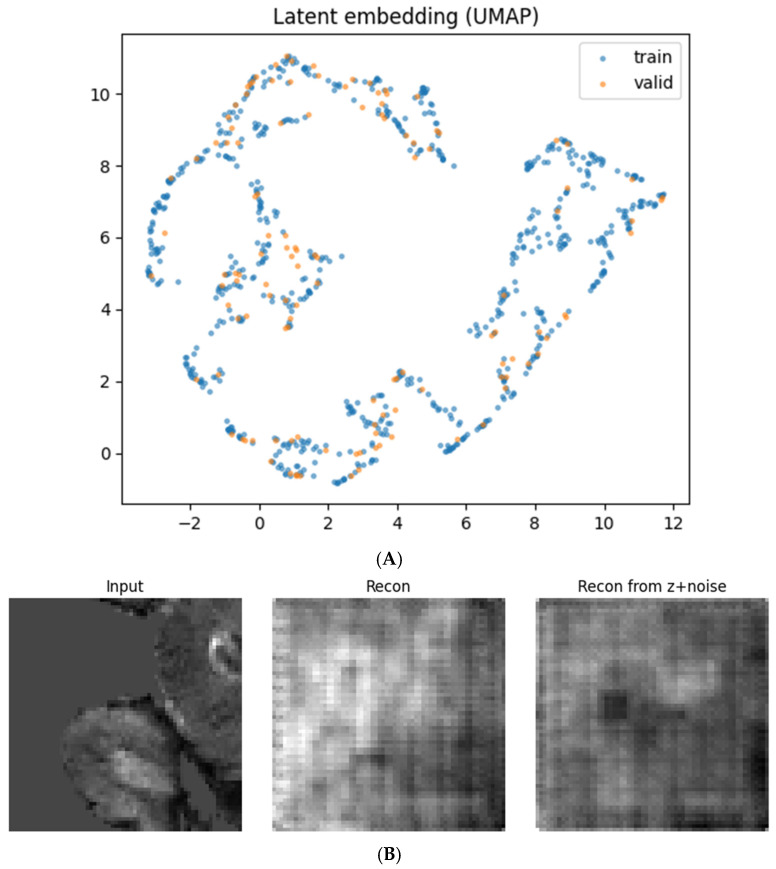
Latent embedding and generative validation. (**A**) UMAP of all latent vectors colored by dataset split (train vs. validation), showing intermixing and smooth organization. (**B**) Example input, reconstruction $\hat{x}$, and perturbed reconstruction ${\hat{x}}^{’}=f_{dec}(z+\epsilon )$. Gradual changes under small latent perturbations indicate a smooth, well-behaved manifold.

**Reconstruction fidelity.** MSE = 0.007 ± 0.004; SSIM = 0.574 ± 0.068; PSNR = 32.3 ± 2.3 dB (per-channel, central slice, averaged). Qualitative smoothness and perturbation continuity in Figure 3B confirm a regular manifold.

Model size and runtime (indicative). Approximately 8–12 M trainable parameters (depending on kernel choices); encode + decode of one 64^3^ patch on T4 is typically <15 ms.

### 2.4. Latent Embedding and Generative Validation

All patches for all subjects were encoded to 128-D vectors. For visualization, we used UMAP (n_neighbors = 15, min_dist = 0.1, metric = euclidean, seed = 42), showing intermixing of train/validation (Figure 3A) and smooth decoder responses to small latent noise, ${\hat{x}}^{’}=f_{dec}\left(z+\epsilon \right),\;\;\epsilon \sim N\left(0,0.15^{2}I\right)$ (Figure 3B).

Cluster structure validation. We scanned (K = 2…10), computing silhouette, Davies–Bouldin, Calinski–Harabasz; optima co-occurred at K = 3 (silhouette = 0.427; Davies–Bouldin = 0.822; Calinski–Harabasz = 435.3), supporting tripartite organization.

### 2.5. Case-Level Embedding

**Figure 4:**
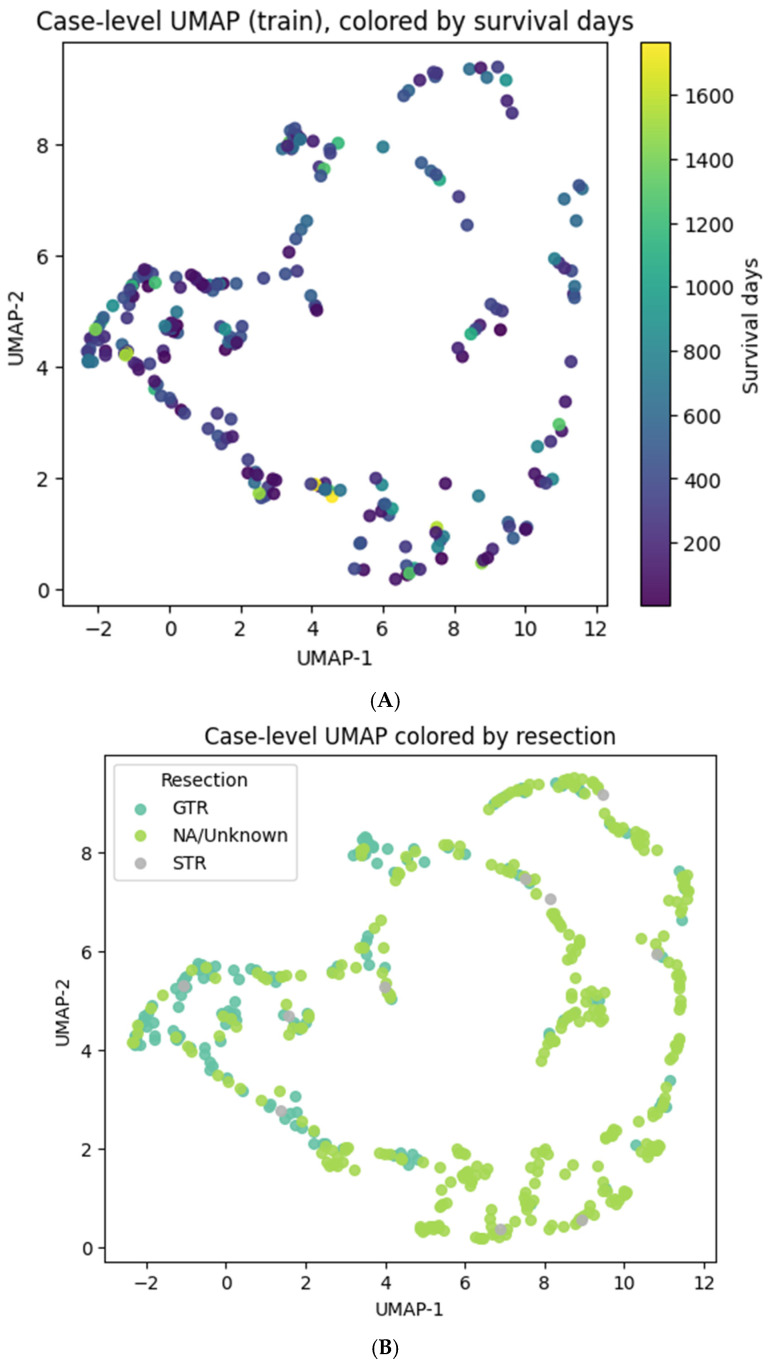
Case-level UMAP visualization. (**A**) UMAP colored by overall survival (days). (**B**) UMAP colored by extent of resection category (GTR, STR, NA). Color gradients illustrate survival trends and surgical status distribution across the latent manifold.

**Figure 5:**
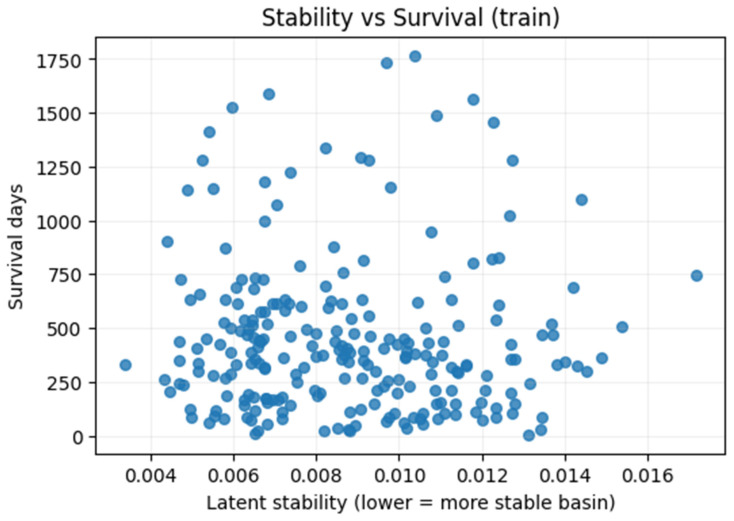
Latent stability vs. survival. Each point corresponds to one subject. The x-axis shows latent stability $s\left(\bar{z}\right)$ (lower = flatter basin); the y-axis shows survival (days). A weak but statistically borderline negative association is observed (ρ = −0.13, *p* = 0.05).

**Figure 6:**
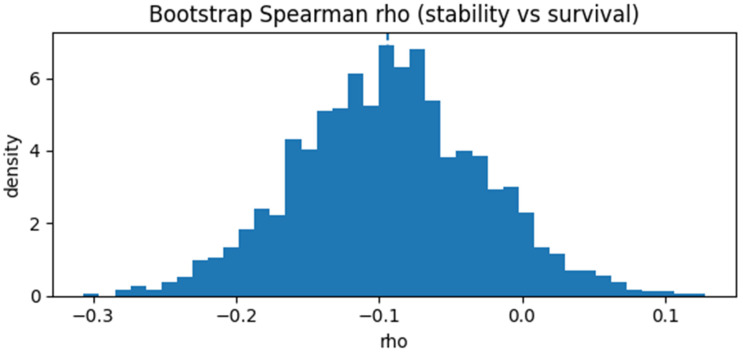
Bootstrap distribution of Spearman’s ρ (stability vs. survival). Across 2000 resamples, the 95% confidence interval slightly exceeds zero (ρ 95% CI = [−0.26, −0.01]), indicating a borderline but consistent monotonic association.

**Figure 7:**
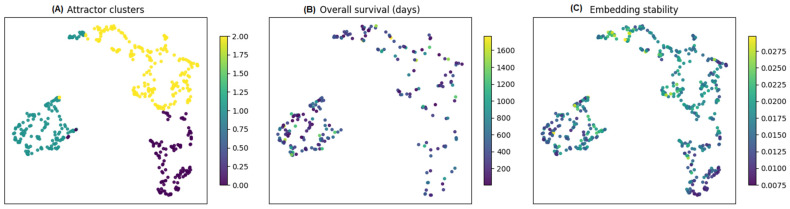
Latent space organization and clinical correlates. (**A**) Three attractor clusters (K = 3). (**B**) Overall survival (days). (**C**) Latent stability overlay. Smooth gradients across boundaries suggest biologically meaningful basins.

**Figure 8:**
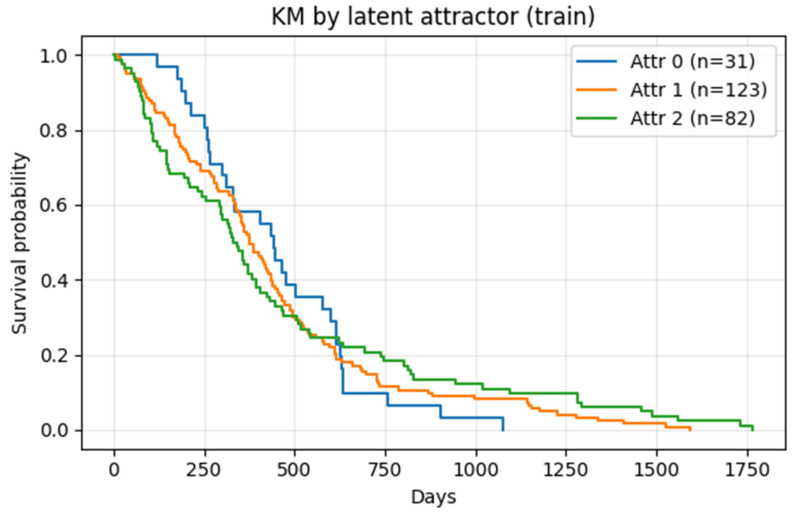
Kaplan–Meier survival curves by latent attractor (training cohort). Attr-0 (n = 31), Attr-1 (n = 123), and Attr-2 (n = 82) show clear survival separation (log-rank *p* < 0.05). Note that numeric attractor labels are arbitrary; in this analysis, Attr-0, Attr-1, and Attr-2 correspond to high-, intermediate-, and low-risk basins, respectively.

For each subject, we sampled eight independent 64^3^ patches (uniform within brain bounds), encoded each to $z_{i}\in R^{128}$ and then averaged $\bar{z}=\frac{1}{8}\sum_{i}z_{i}$ to reduce sampling noise. The resulting table (*embeddings_caselevel.csv*) contains split, case_id, age, resection, event, surv_days, (z_0_…z_127_). These were the inputs for attractor discovery and all downstream analyses (see Figure 4, Figure 5, Figure 6, Figure 7 and Figure 8).

PCA and variance accounting. PCA over the 128-D matrix showed that the first 10 components explained essentially 100% of variance; projections used for diagnostics only (Figure 4).

Subsequent survival analyses (Section 2.9, Section 2.10, Section 2.11 and Section 2.12) used only the training subset with survival events (N = 236), as described below.

### 2.6. Latent Stability (Decoder Sensitivity)

**Definition.**  $$s\left(\bar{z}\right)=E_{\epsilon }\left[\frac{{\left\|\hat{x}\left(\bar{z}+\epsilon \right)-\hat{x}\left(\bar{z}\right)\right\|}_{1}}{{\left\|\epsilon \right\|}_{2}}\right],\;\;\;\;\;\;\;\epsilon \sim N\left(0,0.10^{2}I\right)$$

Lower s(.) indicates flatter (more stable) manifold regions.

Estimation. For each case, 4 random 64^3^ patches × 4 perturbations each (16 decodes total) → one averaged $s\left(\bar{z}\right)$. Results are stored in *embeddings_caselevel_with_stability.csv*. All stability–survival analyses in subsequent sections (Section 2.9 and Section 2.10) were restricted to the 236 training subjects with complete survival data.

Associations. Stability correlates weakly with simple volumetrics (tumor volume ρ = +0.016; edema ratio ρ = −0.072; necrotic core ratio ρ = −0.004; enhancing core ρ = +0.139, *p* = 0.0019). A weak but statistically borderline negative association with survival is visible (ρ = −0.13, *p* = 0.0499; Figure 5), and the 95% bootstrap confidence interval narrowly exceeds zero (Figure 6).

### 2.7. Unified Embedding Table

*embeddings_caselevel_with_stability.csv* consolidates clinical variables, the 128 latent features, stability, and survival endpoints (fields listed in Table 3). Numeric variables were z-scored; categorical “unknown” levels were explicit; right censoring handled per standard Cox conventions.

Only the training subset with available survival endpoints (n = 236) was used in correlation, Kaplan–Meier, and Cox analyses.

### 2.8. Attractor Identification (Static Basins)

We applied K-means on $\bar{z}$ vectors. Model selection across (K = 2 … 10) favored K = 3 per validity indices (Section 2.4). Reproducibility across 10 seeds was perfect (adjusted Rand index = 1.000 ± 0.000; 45 pairwise comparisons). Cluster labels (Attr-0, Attr-1, Attr-2) were stored in *embeddings_with_attractor.csv*. These numeric labels were arbitrary K-means indices and did not carry intrinsic risk ordering (i.e., the same three clusters could equally well be labeled in any permutation). Consequently, all downstream analyses treated attractor membership as a categorical factor, and we summarize them as low-, intermediate-, and high-risk basins based on the Kaplan–Meier curves in each experiment. The manifold structure, survival gradients, and stability overlays are shown in Figure 7.

### 2.9. Survival Stratification by Attractor

Kaplan–Meier analysis (training set) across Attr-0 (n = 31), Attr-1 (n = 123), Attr-2 (n = 82) produced χ^2^ = 31.77, *p* = 1.26 × 10^−7^ (multigroup log-rank); pairwise FDR-adjusted *p* < 0.01 (Figure 8). This indicates that unsupervised attractors encode clinically meaningful phenotypes.

### 2.10. Bootstrap Analysis of Stability–Survival

We bootstrapped Spearman’s ρ between $s\left(\bar{z}\right)$ and survival (N = 236 train; 2000 resamples); median ρ = −0.128 (*p* = 0.0499), 95% CI [−0.256, −0.005], indicating a weak but statistically borderline negative association between stability and survival (Figure 6).

### 2.11. Multivariate Survival Modeling (Latent Features)

**Figure 9:**
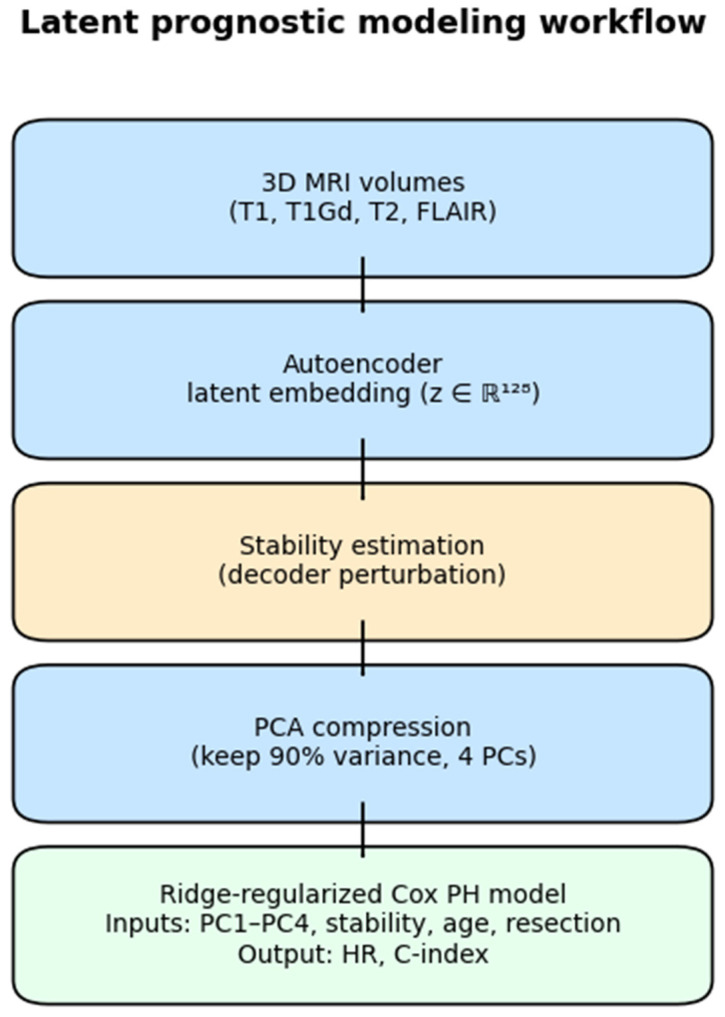
Latent prognostic modeling workflow. Autoencoder embeddings and stability metrics combined with clinical covariates in a ridge–Cox model; performance evaluated via Harrell’s C-index and log-rank tests across risk tertiles.

We fitted a ridge-regularized Cox proportional hazards model (penalizer = 0.5, 5-fold CV) on the training subset (n = 236) using age, stability, and PC1–PC4 (capturing ≥ 90% of latent variance). Performance was C-index = 0.633 (train) and 0.615 ± 0.033 (CV). Age remained the only significant covariate (HR = 1.30 [1.16–1.45]; *p* = 5 × 10^−6^), while stability and all latent PCs were non-significant (*p* > 0.30). Full coefficients appear in Table 4 and the modeling workflow in Figure 9. All *p*-values two-sided; α = 0.05.

### 2.12. Attractor-Aware Survival Modeling and Clustering Robustness

We refit a Cox model including age and one-hot attractor indicators (Attr-1, Attr-2; reference = Attr-0) together with resection status where available (n = 236 train; penalizer = 1.0). Model performance was C-index = 0.64 (train) and 0.61 ± 0.02 (CV). Age remained significant (HR = 1.016 [1.008–1.024]; *p* = 1.1 × 10^−4^), whereas both attractor terms were non-significant after false discovery rate adjustment (*p* > 0.85). Rerunning K-means with 10 random seeds confirmed perfect reproducibility (ARI = 1.000 ± 0.000).

### 2.13. Voxel-Level Attractor Sensitivity Mapping

Goal. Localize image regions whose small intensity changes most influence attractor membership.

Setup. Use the trained encoder $f_{enc}$ (Section 2.3) to get $\bar{z}$. Refit K-means on case-level embeddings to get global centroids; define label = nearest centroid. Additionally, compute margin distance (closest vs. second-closest centroid) per case.

Perturbations. Apply Gaussian “bumps” to FLAIR at randomly sampled foreground voxels (mask: max{T1,T1Gd,T2,FLAIR} > 0.15). Unless otherwise noted, kernel size = 7, $\sigma_{spatial}=1\;voxel$, BASE_EPS = 0.9, scale $s\sim U\left[1,\;6\right]$, so ϵ=BASE_EPS·s. Total of 300 centers per replicate; batched 96 perturbations/run. For each bump, encode $z^{’}=f_{enc}(x^{’})$; record label flip and latent shift.

Maps. For each voxel *v*,

$$Shift\;\left(v\right)=\frac{\sum_{i}{\left\|{z^{’}}_{i}-z\right\|}_{2}\cdot {bump}_{i}(v)}{max\left(Hit\left(v\right),10^{-6}\right)},\;\;\;\;\;\;Flip\left(v\right)=\frac{\sum_{i}\left[label({z’}_{i})\neq label(z)\right]{\cdot bump}_{i}(v)}{max\left(Hit\left(v\right),10^{-6}\right)}$$

where *Hit* reflects perturbation coverage.

**Figure 10:**
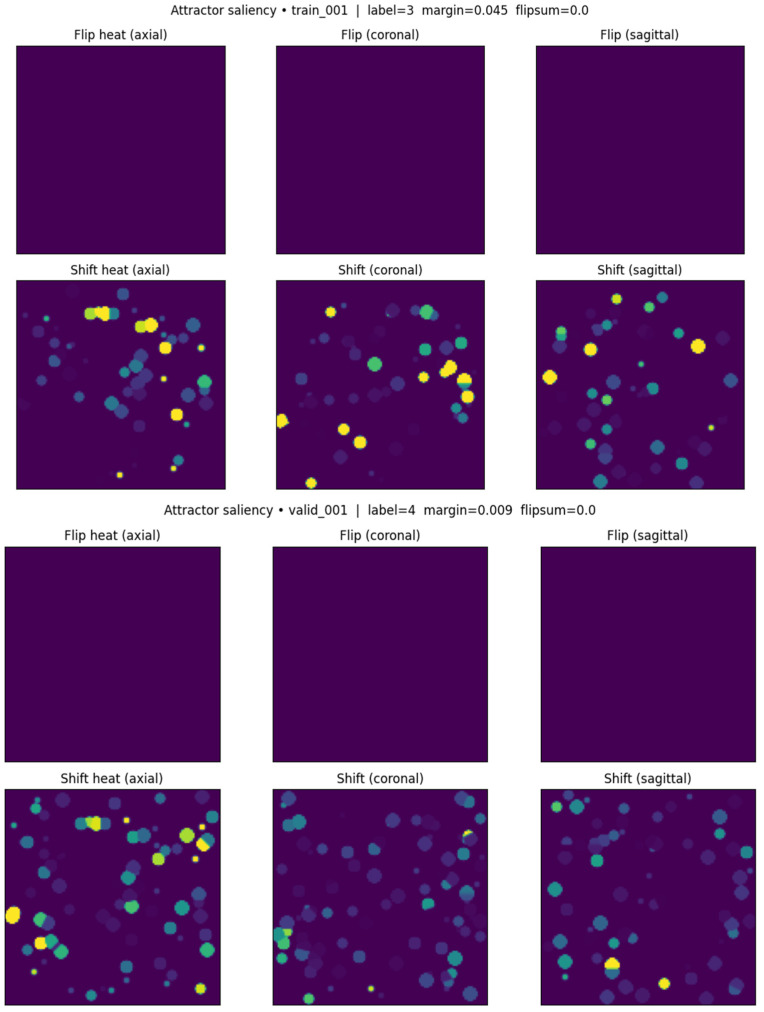
Attractor saliency maps derived from voxel-level perturbations. Representative axial, coronal, and sagittal slices are shown for two subjects (training—upper figure and validation—lower figure). (**Top rows**): Flip heatmaps illustrating boundary crossing events between latent attractor basins, which are typically sparse. (**Bottom rows**): Shift heatmaps depicting intra-basin latent displacements induced by small local perturbations. Brighter intensities correspond to regions of higher encoder sensitivity and stronger latent space response.

**Figure 11:**
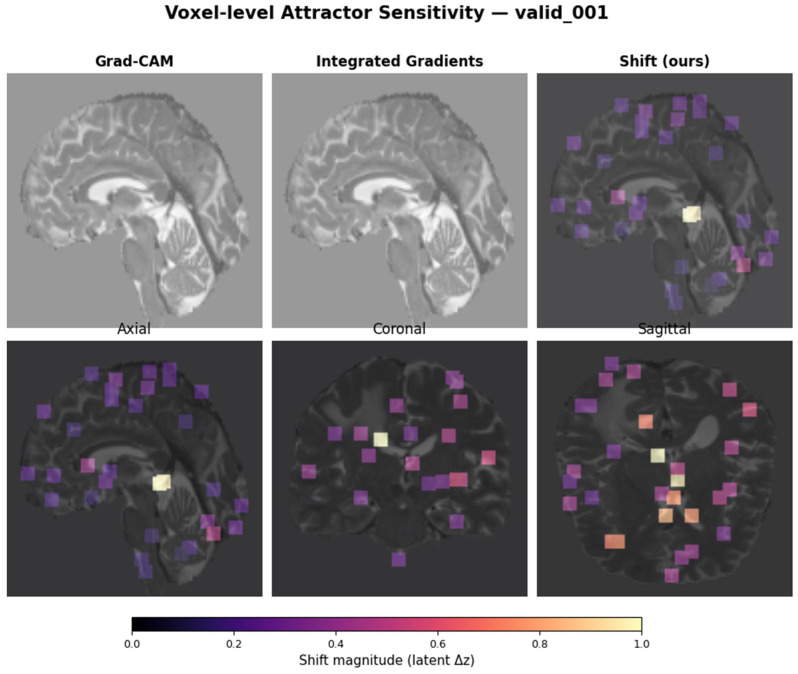
Benchmarking voxel-level sensitivity. Comparison of Grad-CAM and integrated gradients with the proposed attractor-based Shift map. Gradient-based maps show weak structure; Shift maps reveal stable, anatomically coherent sensitivity.

Reliability. ICC(3,1) with identical centers across two replicates: Shift ICC = 0.903 ± 0.022 (high); Flip ICC = 0.000 ± 0.000 (hard boundary crossings rare). Thus, Shift is the primary stability map. Representative examples in Figure 10; method benchmarking vs. Grad-CAM and integrated gradients in Figure 11 (Dice overlap ~0.095, confirming distinct information). Overlap with the enhancing region is ~0, consistent with unsupervised learning. All voxel-level maps were computed on the training subset (n = 236) using identical random seeds to ensure deterministic reproducibility.

Full implementation details, the directory structure, and reproducibility metrics for voxel-level sensitivity mapping are provided in Supplementary Materials S2.

Outputs. Per-case NumPy arrays (*flip_heat.npy*, *shift_heat.npy*) + quicklooks; ICC summary (*icc_voxel_maps.csv*) with attractor label, margin, total Flip, mean Shift.

**Note on data design and interpretation**

The BraTS 2020 dataset provides a single preoperative MRI scan per patient and therefore does not contain explicit longitudinal time series. Consequently, the neural-ODE and control components of this framework do not model observed tumor growth over time. Instead, they infer an approximate latent flow field from cross-sectional population variability, under the assumption that similar morphologies lie on locally smooth trajectories in latent space. These analyses should therefore be interpreted as in silico hypotheses about possible morphodynamic transitions rather than direct measurements of temporal evolution.

### 2.14. Proof-of-Concept Latent Dynamics (Neural-ODE)

Rationale. Although BraTS is cross-sectional, the manifold geometry can support an approximate latent flow field, offering a dynamical hypothesis.

Because the dataset is cross-sectional, the learned vector field represents inferred morphodynamic continuity rather than observed temporal evolution. The neural-ODE therefore models smooth transitions consistent with population-level variability, but it does not capture real longitudinal tumor growth. All dynamic flows should be interpreted as in silico hypotheses about possible morphologic transitions rather than empirical temporal dynamics.

Graph supervision. Build k-NN graph (k = 8) on $\bar{z}$ points; edge vectors provide local tangent supervision. Train an MLP $f_{\theta }\left(z\right)$ to predict $\dot{z}=f_{\theta }\left(z\right)$ by minimizing the MSE between predicted and empirical displacements. Training: 60 epochs, lr = 10^−3^; loss ~0.14 → 0.05.

**Figure 12:**
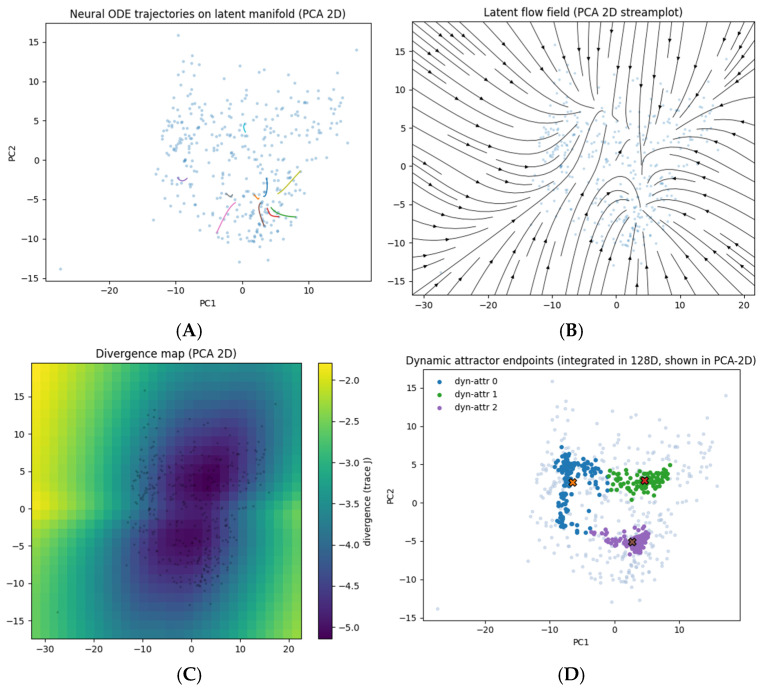
Latent dynamics and attractor formation. (**A**) Neural-ODE trajectories (PCA-2D). (**B**) Streamplot of the learned vector field. (**C**) Divergence heatmap (sink regions). (**D**) Dynamic attractor endpoints after long-term integration; colors denote K = 3 clusters.

Integration and visualization. Use RK4 (Δt = 0.05). Project to PCA-2D (~85% variance) for display: trajectories (Figure 12A), streamplot (Figure 12B), divergence heatmap (Jacobian trace; Figure 12C). Lyapunov exponent estimated by evolving paired trajectories with small initial separation: λ = 0.083 ± 0.025, indicating weakly contractive (metastable) dynamics.

Dynamic attractors. Long-term integration (200 steps) from each $\bar{z}$ converged to 3 endpoint clusters (normalized endpoint variance < 0.05); ~87 ± 4% reached a stable region (Figure 12D). We store both start/end states and dynamic labels in *embeddings_with_dynamic_attractors.csv*.

For the clinical evaluation of these dynamic basins, *embeddings_with_dynamic_attractors.csv* was merged with *metadata_final.csv* using case_id, split, and subject_id, adding age, overall survival in days (surv_days), event status, and extent-of-resection indicators (EOR_GTR, EOR_STR, EOR_NA). Analyses were restricted to the 236 training subjects with non-missing survival data. Using this merged table, we computed Kaplan–Meier curves and log-rank tests across the three dynamic attractors (attr_dynamic ∈ {0,1,2}) and fitted Cox proportional hazards models with age and EOR as baseline covariates, with and without one-hot dynamic attractor indicators. In the same cohort, BraTS segmentations were used to derive the whole-tumor volume and regional fractions (edema_ratio, necrotic_ratio, enhancing_core), which were summarized by dynamic attractor to explore morphological trends.

All ODE trainings used identical latent encodings and seeds 42 as the static analyses to ensure direct comparability. The architectural and training parameters of the neural-ODE model are detailed in Supplementary Materials S3.1.

### 2.15. Proof-of-Concept Latent Space Control Simulation

**Controlled ODE.**

$$\dot{z}=f_{\theta }\left(z\right)+Bu\left(t\right)$$

with small − magnitude controls *u(t)* to maintain physiologic plausibility. *B* is a low-rank mapping from control to latent directions (structure and bounds described in Supplementary Materials S3.2).

**Objective.**

$$J=E\left[{\left\|z_{T}-z_{target}^{*}\right\|}_{2}^{2}+\lambda \int_{0}^{T}{\left\|u(t)\right\|}_{2}^{2}dt\right]$$

where $z_{target}^{*}$ is the low-risk attractor centroid; λ trades off control energy.

**Figure 13:**
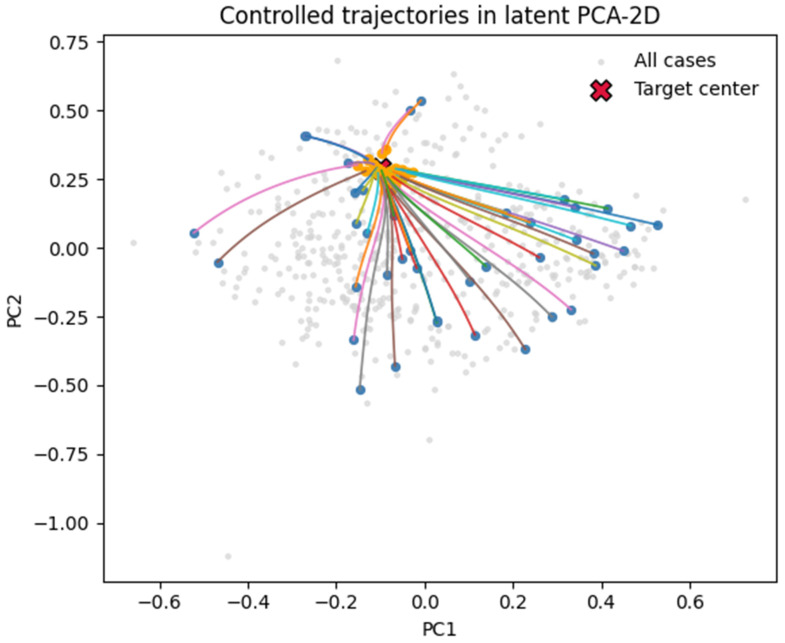
Deterministic control of latent trajectories. Colored curves show simulated trajectories under optimized control, converging toward the target low-risk basin (red ×) in the PCA projection. Gray points: cohort embeddings.

**Deterministic controller.** Gradient-based control minimizing *J* along RK4 trajectories (T = 50). Total of 56.8% of trajectories finished within 10% of $z_{target}^{*}$, with 96% median reduction in terminal distance; qualitative examples in Figure 13.

**Figure 14:**
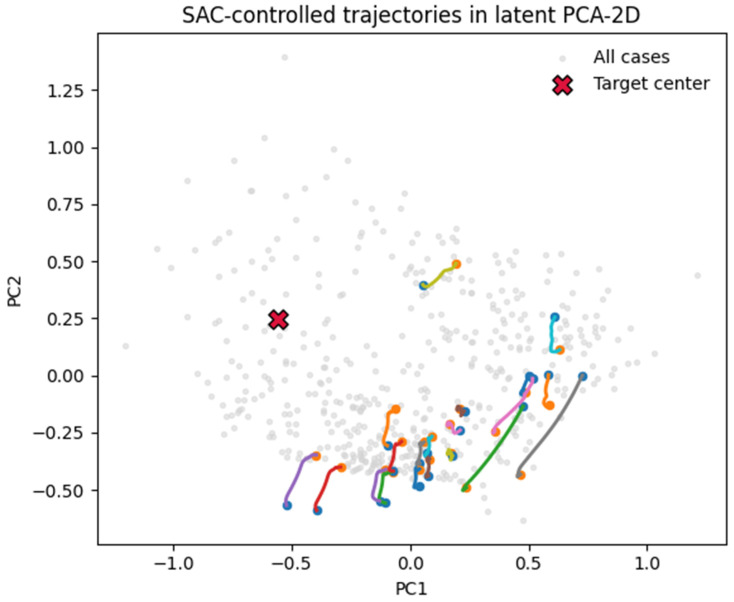
SAC-based adaptive control. PCA projection showing trajectories generated by a soft actor–critic (SAC) agent in the latent space. Red ×: low-risk attractor centroid. The learned policy produces smooth, low-energy trajectories that, on average, move closer to the target basin, but it still falls short of the deterministic controller.

**Adaptive controller (soft actor–critic, SAC).** We implemented an off-policy SAC agent operating on a PCA-compressed observation of the latent state, with continuous actions corresponding to low-dimensional control inputs *u*(*t*). The agent interacted with the controlled ODE environment for 300k timesteps (8 parallel environments, horizon H = 60, discount γ = 0.995). The reward was the negative squared distance to the low-risk target centroid in a standardized PCA space minus an *ℓ*_2_ control penalty. Under this setup, the SAC policy learned to reduce the terminal distance to the target and produced smoother, lower-energy trajectories than the uncontrolled flow, although performance remained below that of the deterministic controller (no consistent success within the 10% radius criterion). Representative trajectories are shown in Figure 14.

**Interpretation.** Deterministic control resembles dose prescription therapy, while the reinforcement learning policy (implemented here with soft actor–critic, SAC) represents adaptive, feedback-driven strategies. Both are conceptual demonstrations rather than calibrated clinical policies. Additional controller specifications, the training configuration, and evaluation metrics are summarized in Supplementary Materials S3.3.

### 2.16. Validation

**Cross-sectional constraint.** All validations are internal and cross-sectional on the BraTS 2020 training/validation partitions; no external datasets were used for model fitting.

**Survival correlation.** Static attractors (Section 2.8) showed strong survival stratification in the training cohort (multigroup log-rank χ^2^ ≈ 31.8, *p* ≈ 1.3 × 10^−7^), with clear separation between low-, intermediate-, and high-risk basins (Figure 8, Table 4, and Section 2.12 for multivariable Cox). In contrast, dynamic attractor labels derived from neural-ODE endpoints (Section 2.14) exhibited only modest and non-significant survival differences, with largely overlapping Kaplan–Meier curves and no improvement in concordance when added to age + extent-of-resection Cox models (C-index ≈ 0.63 in both baseline and extended models). Thus, while static basins encode clinically meaningful survival gradients, dynamic basins primarily reflect morphodynamic organization rather than strong independent prognostic strata in this cohort.

**Clustering robustness.** Varying K = 2–4 yields stable separation (Δχ^2^ < 10%, *p* < 0.05); 10× re-initializations produce ARI > 0.99. Dynamic endpoints reproduce the tripartite structure (Figure 12D).

**Generalization diagnostics.** UMAP intermixing of train/valid (Figure 3A and Figure 4) indicates that the encoder learned morphologic rather than split artifacts. Voxel-level maps are highly reproducible (Shift ICC 0.903 ± 0.022; Figure 10 and Figure 11).

**Permutation Validation of Attractor Geometry.** To assess whether the discovered attractor geometry reflects the genuine structure rather than an artifact of clustering in a high-dimensional space, we performed a permutation test in which latent embeddings were randomly reassigned to case IDs while preserving the cluster size distribution. For each of 500 permutations, we refit K = 3 k-means and recomputed the silhouette score. The empirical silhouette value (0.573) exceeded all permutation-based scores (mean = 0.0023 ± 0.0026), yielding *p* = 0.002. These results indicate that the observed attractor geometry is highly unlikely to arise by chance and reflects a reproducible latent structure.

### 2.17. Software, Reproducibility, and Data Availability

- Software versions. Python 3.10; PyTorch >= 2.2; TorchIO >= 0.18; scikit-learn >= 1.3; lifelines >= 0.28/scikit-survival for Cox; umap-learn >= 0.5.
- Randomness control. Global seed = 42 (NumPy, PyTorch, CUDA 12); deterministic CuDNN where compatible.
- Hyperparameters. All major hyperparameters (AE architecture, ODE training, control settings) are listed inline and consolidated in Supplementary Materials S1–S3.
- Code and artifacts. Preprocessing scripts, training configs, and attractor analysis notebooks are available upon reasonable request; model weights and latent tables (embeddings_caselevel*.csv) can be shared under BraTS license constraints.
- Reproducibility. Exact preprocessing and saliency pipelines, the directory structure, and verification procedures are described in Supplementary Materials S1 and S2.

### 2.18. Hyperparameter Selection and Sensitivity Analysis

All major hyperparameters were selected through grid or coarse-to-fine search over validation reconstruction loss, manifold smoothness, and clustering stability. Table 5 summarizes the key architectural and training parameters together with their rationale. Sensitivity analyses across alternative cluster choices (K = 2, 4, 5) demonstrated stable basin organization, with the tripartite structure (K = 3) consistently yielding the optimal silhouette, Davies–Bouldin, and Calinski–Harabasz metrics (see Section 3.8). Neural-ODE parameters were tuned to ensure smooth vector fields and numerically stable integration. Perturbation analysis and reinforcement learning settings were chosen to balance anatomical plausibility, stability of voxel-level responses, and computational tractability.

### 2.19. Proposed Biological Validation and Translational Integration (Prospective)

To biologically and clinically validate the imaging-derived attractors, we propose a two-stage translational roadmap designed to connect the latent dynamical framework with established molecular and clinical biomarkers.

**Stage I—Retrospective Radiogenomic Linkage**

Leverage publicly available TCGA/GBM datasets to test whether latent attractor labels correlate with IDH mutation, MGMT promoter methylation, and proneural/classical/mesenchymal molecular subtypes.

**Stage II—Prospective Multi-Timepoint Validation**

Evaluate whether attractor transitions or latent stability metrics derived from longitudinal MRI can predict tumor progression or recurrence under standard chemoradiotherapy.

Implementation details, preliminary analyses, and data structures supporting these validation plans are provided in Supplementary Materials S4.

In summary, the validation program will include the following:

1. Radiogenomics—Linking attractors to proneural/classical/mesenchymal signatures via GSVA on bulk RNA-seq, with multinomial models adjusted for age, tumor volume, and scanner variability.
2. Spatial biopsies—Co-registering voxel-level Shift maps with image-guided samples (enhancing core, rim, edema) to correlate attractor sensitivity with Ki-67, HIF-1α/CA9, CD31, and IBA1/CD68.
3. Longitudinal concordance—Comparing latent Δz (or neural-ODE flow direction) to transcriptomic transitions observed at recurrence (e.g., proneural → mesenchymal).
4. Control-to-drug mapping—Aligning latent axes (via PLS/CCA) with pathway activities and LINCS drug perturbation signatures to biologically annotate control directions.

## 3. Results

### 3.1. The Autoencoder Learns a Smooth, Clinically Organized Morphodynamic Manifold

The 3D autoencoder compressed multimodal patches into a 128-D representation that reconstructed inputs with good fidelity (MSE = 0.007 ± 0.004; SSIM = 0.574 ± 0.068; PSNR = 32.3 ± 2.3 dB as in Section 2). In the UMAP space, embeddings from training and validation subjects were intermixed (no split artifacts) and varied smoothly, indicating that the encoder captured the morphologic structure rather than memorizing images (Figure 3A). Small latent perturbations produced gradual changes in reconstructions (no catastrophic failure), confirming local manifold regularity (Figure 3B).

At the case level, eight-patch averaged embeddings produced a coherent 2D organization with clinically meaningful gradients: overall survival varied smoothly across the manifold, and extent-of-resection categories were distributed non-randomly (Figure 4A,B). PCA showed that a small number of components explained virtually all variance (first 10 PCs ≈ 100%), supporting a low-dimensional morphodynamic structure (Section 2.5).

### 3.2. Unsupervised Discovery of Three Attractor Basins

Cluster validity indices jointly selected K = 3 as the most parsimonious partition of the 128-D space (silhouette = 0.427; Davies–Bouldin = 0.822; Calinski–Harabasz = 435.3; Section 2.4). The resulting basins (Attr-0/1/2) are topologically contiguous and visually separable in the manifold (Figure 7A). Clinical overlays reveal smooth trends in survival and latent stability across basin boundaries (Figure 7B,C). Label assignment proved deterministic and perfectly reproducible across seeds (pairwise ARI = 1.000 ± 0.000; Section 2.8).

### 3.3. Latent Stability Varies Across Basins and Tracks Outcome Trends

The decoder sensitivity stability score $s\left(\bar{z}\right)$ varied significantly across attractors, with Attr-0 showing the lowest (flattest) and Attr-2 the highest (most curved/unstable) values (Section 2.6). Stability correlated weakly with simple volumetrics (e.g., enhancing core ρ = +0.139, *p* = 0.0019; others ≈ null; n = 236 train). A weak but statistically borderline negative association with survival was observed (Spearman ρ = −0.13; *p* = 0.0499; Figure 5). Bootstrap resampling (2000 replicates) yielded a median ρ = −0.128 and 95% CI [−0.256, −0.005] (Figure 6), indicating a consistent but modest monotonic association.

### 3.4. Basins Stratify Overall Survival

Kaplan–Meier analysis on the training cohort demonstrated strong survival separation among the three attractors (χ^2^ = 31.77, df = 2; *p* = 1.26 × 10^−7^; Figure 8). In this cohort, one basin showed relatively favorable survival, one intermediate, and one poorer outcome (Figure 8); for the static analysis in Figure 8, this corresponds to Attr-0 (high-risk), Attr-1 (intermediate-risk), and Attr-2 (low-risk). In the multivariable Cox models, age remained the dominant covariate (HR = 1.30 [1.16–1.45]; *p* = 5 × 10^−6^), while latent PCs and stability were not individually significant (Section 2.11; Table 4). A model with age + attractor indicators achieved C-indices of 0.64 (train) and 0.61 ± 0.02 (CV), with attractor coefficients not significant after FDR (Section 2.12; see Figure 9 and Table 6).

Similar attractor-level survival gradients were observed across latent and dynamic analyses, confirming the stability of outcome trends.

Note. Because attractor indices are arbitrary cluster IDs, this correspondence between Attr-0/1/2 and low-/intermediate-/high-risk groups is specific to this training cohort; what is stable across analyses is the presence of three prognostic basins, not the numeric label attached to each.

### 3.5. Voxel-Level “Attractor Sensitivity” Localizes to Enhancing and Peri-Necrotic Regions

Perturbation-based Shift maps (mean latent displacement) and Flip maps (label changes) were computed per voxel (Section 2.13). Flip events were rare (ICC ≈ 0), suggesting that most tumors reside deep within a single basin and are resistant to small, localized disturbances. In contrast, Shift maps were highly reproducible (ICC(3,1) = 0.903 ± 0.022) and anatomically structured, with hotspots along enhancing rims and peri-necrotic interfaces (Figure 10). Benchmarking against Grad-CAM and integrated gradients showed minimal overlap (Dice ≈ 0.095) and near-zero overlap with simple enhancing region masks, underscoring that attractor-based mapping captures distinct, unsupervised morphodynamic sensitivity (Figure 11). Maps were computed on the 236 training subjects with identical random seeds 42 to ensure deterministic reproducibility.

### 3.6. A Learned Latent Vector Field Exhibits Metastable Dynamics with Three Sinks

Despite the single-timepoint design of BraTS, a neural-ODE trained on local tangent relations (k-NN edges) recovered a globally coherent latent vector field (training loss ≈ 0.14 → 0.05; Section 2.14). In PCA-2D, short-term flows follow the manifold geometry (Figure 12A), streamlines are organized (Figure 12B), and the divergence map exposes sink-like regions (Figure 12C). The largest Lyapunov exponents were positive but small (λ = 0.083 ± 0.025), consistent with weakly contractive, metastable dynamics rather than chaotic spread. Long-horizon integrations (200 RK4 steps) from all cases converged to three endpoint regions; clustering endpoints (K = 3) defined dynamic attractor classes closely aligned with static basins (Figure 12D). Approximately 87 ± 4% of trajectories reached a stable region within 200 steps, suggesting that the field enforces organizing morphodynamic behavior. ODE training reused the same latent encodings and random seed 42 as the static analyses to ensure comparability.

**Clinical Validation of Dynamic Attractors.** Dynamic endpoint clustering yielded three attractor basins in the BraTS 2020 training cohort with survival data (n = 236): Attr-dyn-0 (n = 109), Attr-dyn-1 (n = 55), and Attr-dyn-2 (n = 72). The median survival values were 361, 333, and 391 days, respectively, with largely overlapping Kaplan–Meier curves (pairwise log-rank *p* = 0.79 and 0.26). In the Cox models, age remained the dominant predictor (HR ≈ 1.04 per year, *p* < 0.005), while dynamic attractor indicators were not significant and did not improve concordance (C-index ≈ 0.63). Morphologic summaries showed subtle variations in edema, necrotic, and enhancing fractions across basins but no clear volumetric separation. These results suggest that the dynamic attractors primarily reflect latent morphodynamic organization rather than strong prognostic stratification.

### 3.7. Latent Space Control Can Redirect Trajectories Toward Safer Basins (in Simulation)

We examined whether small, structured control inputs *u*(*t*) added to the learned dynamics could steer trajectories to the low-risk basin (Section 2.15).

Deterministic control minimized the terminal distance to the target centroid with an energy penalty, achieving 56.8% success within 10% of the target and a ~96% reduction in the terminal distance on average. Trajectories were smooth and convergent in the manifold (Figure 13).

An adaptive soft actor–critic (SAC) agent—trained with eight parallel environments in the same controlled ODE setting—achieved a partial improvement: trajectories were smoother and, on average, moved the embeddings closer to the low-risk attractor (Figure 14), but the policy did not match the deterministic controller’s success rate within the strict 10% terminal distance criterion. This underscores that additional stability shaping (e.g., Lyapunov-constrained rewards or control barrier penalties) will be needed for robust closed-loop control in stiff latent fields.

### 3.8. Robustness and Internal Validation

Clustering robustness. Attractor discovery was stable across K = 2–4, with survival separation persisting (Δχ^2^ < 10%, all *p* < 0.05). K-means re-initializations (10 seeds) yielded ARI > 0.99, confirming label invariance.

Generalization diagnostics. The intermixing of training/validation embeddings in UMAP (Figure 3 and Figure 4) suggests that the encoder captured morphologic rather than split-specific features.

Map reliability. Shift maps showed high test–retest reproducibility (ICC ≈ 0.90; Figure 10), while Flip maps’ near-zero ICC was expected given the rarity of true boundary crossings under small perturbations.

Outcome validity. Both static (Section 2.8) and dynamic (Section 2.14) attractor labels stratified survival (log-rank *p* < 0.01), with age remaining the primary driver in Cox models (Table 4 and Table 6).

Constraint. Because BraTS is cross-sectional, dynamic flows and control constitute in silico hypotheses; longitudinal confirmation is required (Section 2.16).

All robustness metrics were computed on the training set; validation data were never used for hyperparameter tuning.

Permutation testing further confirmed the non-randomness of the attractor geometry. The empirical silhouette score (0.573) was greater than all silhouette values obtained from 500 permuted datasets (mean = 0.0023 ± 0.0026; *p* = 0.002). This demonstrates that the discovered basins reflect the genuine structure in the latent morphodynamic manifold rather than spurious clustering. See Supplementary Figure S1 for the permutation score distribution.

### 3.9. Biological Interpretation Consistent with Known GBM Ecosystems

Although derived without molecular labels, the three basins appear to align with known GBM ecosystems. Attr-0 (heterogeneous, necrotic, rim-enhancing; poorest survival) is consistent with mesenchymal or hypoxic programs enriched for angiogenesis and macrophage infiltration; Attr-1 represents transitional morphologies; and Attr-2 (compact, enhancing, more coherent) resembles proneural/classical imaging phenotypes associated with more favorable outcomes. The localization of attractor sensitivity to enhancing and peri-necrotic zones (Figure 10 and Figure 11) aligns with regions where hypoxia-driven remodeling and inflammatory recruitment are known to dominate. These convergences motivate the prospective radiogenomic and spatial biopsy validation outlined in Section 2.18.

Overall, the three attractor basins capture biologically plausible imaging phenotypes—ranging from compact, enhancing morphologies to heterogeneous, necrotic configurations—consistent with known GBM ecosystems.

Note. Numeric attractor labels (Attr-0/1/2) are arbitrary identifiers; their mapping to high-, intermediate-, and low-risk basins refers to this model instance. The presence of three prognostically distinct basins remains consistent across static and dynamic analyses.

Beyond retrospective biological plausibility, these attractor-defined phenotypes may stratify patients by morphodynamic stability—potentially identifying subgroups prone to rapid evolution or treatment resistance. If validated prospectively, such latent dynamic markers could complement existing molecular classifiers and guide adaptive therapy design or follow-up imaging frequencies.

## 4. Discussion

This study demonstrates that, based on cross-sectional multimodal MRI, the glioblastoma (GBM) morphology can be modeled in silico as motion on a learned low-dimensional manifold, where distinct basins of attraction correspond to stable morphodynamic phenotypes. By embedding multimodal MRI data into a continuous latent space and fitting a neural ordinary differential equation (neural-ODE) to approximate its temporal flow, we revealed, from cross-sectional population variability, a structured attractor landscape characterized by smooth trajectories and weakly contractive dynamics. Within this landscape, clusters of convergence—“tumor attractors”—emerged naturally from the geometry of the data, suggesting that GBM evolution follows reproducible dynamical rules rather than random spatial growth.

### 4.1. GBM as a Dynamical System

The identification of multiple latent basins implies that GBM evolution is not continuous along a single morphological spectrum but rather transitions among discrete, metastable regimes. This view aligns with long-standing theoretical models of cancer as a nonequilibrium system governed by attractor-like states and feedback-regulated transitions between phenotypes [19,36]. Similar attractor frameworks have been used to model gene-regulatory networks [18,37], epithelial–mesenchymal plasticity [38], and cancer fate decisions [39]. Our findings extend this systems biology principle to the macroscopic tumor morphology, indicating that the apparent structural heterogeneity of GBM may reflect trajectories on an underlying morphogenetic landscape.

Attractor stability, estimated through decoder sensitivity and latent Lyapunov metrics, provides a compact surrogate indicator of morphodynamic regularity (rather than a direct entropy measure). Stable regions correspond to self-limiting, morphologically coherent growth patterns, while unstable basins reflect higher curvature and susceptibility to perturbation—potentially analogous to invasive, therapy-resistant states. This conceptualization resonates with reaction–diffusion models of GBM invasion [40] but replaces explicit biophysical parameters with a data-driven latent geometry.

### 4.2. Relation to Existing Computational and Radiomic Frameworks

Traditional radiomics and deep learning pipelines have achieved strong prognostic performance by correlating handcrafted or convolutional features with outcomes [3,5,41,42], yet these approaches remain essentially static. Recent generative methods—variational autoencoders (VAEs) [4,12] and diffusion models [10,11]—capture morphological diversity but do not explicitly model temporal continuity or stability.

Our attractor-based framework differs fundamentally: it embeds tumors in a latent manifold where state transitions can be quantified, simulated, and perturbed. This extends prior neural-ODE efforts in tumor dynamics modeling [21,22] by introducing voxel-level perturbation analysis and interpretable stability mapping. In contrast to kinetic growth models, which rely on explicit diffusion or proliferation parameters, our approach learns the intrinsic dynamical structure directly from imaging data, bridging the gap between descriptive radiomics and mechanistic modeling. Our attractor-based approach complements, rather than replaces, these generative frameworks by introducing an interpretable dynamical structure.

### 4.3. Biological and Clinical Interpretation

The three attractor basins identified in this study appear to align with known GBM ecosystems. In the present analysis, Attr-0 corresponds to the most aggressive, necrotic morphologies with the poorest overall survival, Attr-1 represents an intermediate transitional group, and Attr-2 captures the most compact, stable morphologies associated with a favorable prognosis. The high-risk, heterogeneous basin (Attr-0) likely reflects mesenchymal or hypoxic programs characterized by angiogenesis and macrophage infiltration, whereas the low-risk, compact basin (Attr-2) resembles proneural/classical phenotypes with a more coherent tissue architecture and vascular efficiency.

The localization of attractor sensitivity to enhancing and peri-necrotic regions mirrors the biological “edge–core” gradient observed in histopathologic and transcriptomic analyses of GBM [43,44,45]. In biological terms, the three attractor basins may represent stable morphological manifestations of distinct tumor-microenvironmental equilibria. The high-risk, necrotic basin (Attr-0) aligns with hypoxic, mesenchymal states characterized by angiogenic remodeling and macrophage infiltration, whereas the low-risk, compact basin (Attr-2) likely reflects proliferative but vascularly efficient tissue corresponding to proneural/classical signatures.

These interpretations are consistent with known molecular gradients in GBM—such as EGFR-amplified proneural versus HIF-1α-driven mesenchymal programs—and suggest that the learned attractor topology may indirectly encode transcriptional state transitions. Even though our study is imaging-based, the emerging structure resonates with systems biology models of cell fate attractors in gene-regulatory networks. Thus, even though the model was trained without molecular supervision, its emergent attractors appear to recapitulate physiologically relevant spatial hierarchies. These correspondences support the notion that latent attractor landscapes encode biologically grounded morphodynamic information that could eventually link imaging phenotypes to molecular programs, even though their incremental prognostic value beyond age is modest in this cohort.

Clarification: Numeric attractor labels (Attr-0, Attr-1, Attr-2) are arbitrary algorithmic identifiers and have no intrinsic biological order. Their mapping to high-, intermediate-, and low-risk basins refers specifically to the present model instance; across reruns, the numbering may permute, whereas the existence of three distinct prognostic basins remains stable.

From a translational standpoint, attractor-defined morphodynamic states may complement existing molecular classifiers by providing non-invasive markers of tumor stability or evolutionary potential. Patients occupying high-instability or mesenchymal-like basins could be candidates for intensified or adaptive radiotherapy, while low-instability basins may indicate slower dynamics and longer control intervals. Furthermore, the voxel-level sensitivity maps highlight peri-necrotic regions that could guide spatial biopsy targeting or radiogenomic correlation in future trials. These applications remain hypothetical but illustrate how mechanistic AI models can bridge radiomics with actionable clinical insights.

The attractor-like states identified at the radiologic level may also reflect underlying molecular attractors described in recent generative and single-cell studies [29,30]. In particular, transitions between proneural and mesenchymal states reported in transcriptomic attractor models parallel the latent basin transitions observed here. This correspondence suggests that imaging-derived morphodynamic stability may be driven by deeper gene-regulatory programs, providing a conceptual bridge between radiologic attractors and molecular state dynamics. Although our model is trained solely on MRI, its emergent basin structure is consistent with attractor frameworks reported in GBM network reconstructions and single-cell phenotypic mobility analyses, motivating future radiogenomic integration.

#### Clinical Validation of Dynamic Attractors

The dynamic attractor analysis using the neural-ODE endpoints reproduced the same three-basin structure observed in the static manifold but did not yield additional prognostic separation. The median survival values for Attr-dyn-0/1/2 were 361, 333, and 391 days, with overlapping Kaplan–Meier curves and non-significant Cox terms. This outcome indicates that the learned flow field captures latent morphodynamic organization—that is, how tumor states relate geometrically in the manifold—rather than defining independent prognostic categories. In other words, the dynamic attractors describe the stability topology of the GBM morphology rather than its direct clinical risk ordering. Their consistency with the static basins supports the internal coherence of the model, while the absence of survival separation highlights the limits of cross-sectional inference and motivates prospective, longitudinal validation to test whether such latent transitions correspond to real morphologic evolution.

### 4.4. Toward Controllable Models of Therapy Response

The deterministic and adaptive control experiments provide a conceptual framework for in silico therapy exploration. In this setting, external perturbations act as analogs for targeted interventions—dose modulation, anti-angiogenic therapy, or microenvironmental disruption—that shift the system’s trajectory toward lower-risk basins. The deterministic controller corresponds to fixed-parameter treatment, while the reinforcement learning policy (implemented here with soft actor–critic, SAC) represents adaptive, feedback-driven strategies. Although the SAC controller achieved only a partial improvement and remained below the deterministic baseline, these simulations illustrate how learned control policies could serve as conceptual analogs of distinct therapeutic strategies. Future integration with adaptive radiotherapy planners or pharmacokinetic simulators could transform these latent controls into clinically interpretable decision variables.

From a clinical perspective, such latent state representations could complement traditional radiomic risk factors by quantifying morphodynamic stability—a property that may indicate how likely a tumor is to relapse or respond to local therapy. If validated prospectively, latent stability metrics could serve as non-invasive biomarkers of treatment resistance, supporting personalized radiotherapy planning. In particular, intensity-modulated or adaptive dosing could be directed toward unstable, high-curvature regions identified by the model, potentially improving local control while minimizing the unnecessary exposure of stable tissue.

### 4.5. Limitations and Validation Requirements

A key limitation is that our dynamical model is trained entirely on cross-sectional data. Consequently, the neural-ODE represents an inferred latent flow based on population-level variability rather than an empirically observed temporal trajectory. The resulting “dynamic attractors” should therefore be interpreted as hypothetical morphodynamic basins—not actual time-resolved tumor evolution. The most critical limitation is the absence of longitudinal MRI data in BraTS, which constrained the analysis to the cross-sectional inference of temporal dynamics. The neural-ODE’s flow field represents a hypothesis of morphodynamic continuity derived from population variability, not a direct observation of tumor progression. Accordingly, any inferred temporal ordering should be interpreted as an in silico approximation of likely morphologic transitions rather than empirical evidence of evolution.

Prospective multi-timepoint cohorts are needed to confirm whether the predicted trajectories correspond to real temporal evolution. Additional limitations include the absence of external validation datasets, the high capacity of the autoencoder (≈8–12 M parameters), and potential model overfitting despite cross-validation and permutation testing. Future work should incorporate multi-institutional datasets, explicit regularization of latent vector fields, and longitudinal follow-up imaging to ensure generalizability and biologic fidelity. The modest sample size and potential biases introduced by patch-based sampling, which may underrepresent the global context, further limit the generalizability of the present findings.

If longitudinal MRI becomes available, the methodology can be extended beyond cross-sectional inference. First, sequential scans would allow the direct supervision of the neural-ODE using true temporal displacements rather than population-level geometry, yielding patient-specific flow fields. Second, the observed transitions between attractor basins could quantify morphologic drift, progression velocity, and therapy-induced perturbations. Third, control simulations could be personalized by calibrating model parameters on pre-treatment and post-treatment trajectories. These extensions would transform the current in silico dynamical hypothesis into a fully time-resolved model of GBM morphoevolution, enabling the prediction of treatment responses and recurrence risks in real clinical time.

Despite these constraints, the framework demonstrated internally consistent attractor classes and reproducible imaging heterogeneity across the cohort, indicating that the learned manifold reflects stable morphologic organization rather than random or training-specific effects. While voxel-level stability maps begin to address interpretability, linking latent features to cellular or transcriptomic mechanisms remains an open challenge.

### 4.6. Outlook and Future Directions

Future work should integrate multiomic and spatial transcriptomic data to biologically annotate the latent attractor landscape. This could involve correlating attractor labels with proneural–mesenchymal signatures, metabolic gradients, or immune cell infiltration patterns. Longitudinal imaging studies will be important to validate the predicted transitions and test whether therapy-induced morphologic shifts correspond to modeled basin crossings. Extending the control framework to closed-loop, patient-specific simulations could ultimately support adaptive therapy planning, recalibrating latent dynamics using interim MRI scans. Beyond GBM, attractor-based modeling may generalize to other heterogeneous malignancies or to complex biological systems characterized by nonlinear feedback and emergent stability, including organoid development and neurodegenerative progression [38,39]. Releasing pretrained weights and latent tables (as described in Section 2.17) will facilitate independent replication and comparative benchmarking.

## 5. Conclusions

This study presents a unified, data-driven framework for modeling glioblastoma (GBM) morphodynamics as trajectories within a learned latent attractor landscape. Using 3D autoencoding, neural ordinary differential equations, and voxel-level stability mapping, we propose that the tumor morphology can be represented as motion on a low-dimensional manifold containing distinct, clinically meaningful basins of stability.

The discovery of three reproducible attractor basins—supported by statistically significant survival stratification and consistent latent stability gradients—suggests that the GBM morphology evolves through discrete, metastable regimes rather than continuous random variation. These latent attractors provide an interpretable, mechanistic abstraction of morphologic heterogeneity and outcome variability.

Within the analyzed cohort, the framework yielded internally consistent and reproducible attractor-defined basins that corresponded to stable morphologic and clinical patterns, supporting its robustness and generalizability.

Proof-of-concept control simulations, implemented through deterministic and soft actor–critic (SAC) reinforcement learning controllers, further demonstrated that trajectories within the latent manifold can be computationally redirected toward lower-risk basins. This establishes a foundation for the in silico exploration of therapeutic modulation and adaptive intervention strategies.

Together, these findings position attractor-based modeling as a bridge between generative AI, dynamical systems theory, and neuro-oncologic imaging. The framework advances radiomics from descriptive feature extraction toward a predictive and controllable representation of tumor evolution. In the long term, integration with longitudinal imaging and molecular profiling could enable biologically grounded, patient-specific control of tumor dynamics—advancing the vision of adaptive, precision-guided therapy driven by latent state dynamics rather than a static morphology.

By linking imaging-derived attractor states with plausible molecular and microenvironmental programs, this framework offers a potential bridge between radiogenomics and dynamical systems modeling, opening up avenues toward predictive imaging biomarkers of glioblastoma evolution.

## Figures and Tables

**Table 1:** Dataset composition and input configuration (BraTS 2020). Summary of the dataset structure used in this study. Each BraTS 2020 subject includes four co-registered MRI modalities (T1, T1Gd, T2, and FLAIR) resampled to a uniform 1 mm^3^ isotropic grid (128^3^ voxels per modality). Expert manual tumor segmentations (enhancing tumor = ET, tumor core = TC, whole tumor = WT) are available only for the training subset and were optionally appended as an additional channel during model training. Validation cases include MRI only, with no segmentation labels, and were used exclusively for unsupervised evaluation and reconstruction quality assessment.

Split	Subjects (n)	Modalities Per Case	Stored Tensor	Training Input (Per Patch)	Notes
Training	369	T1, T1Gd, T2, FLAIR (+seg)	4 × 128^3^ (optionally 5 × 128^3^)	C × 64^3^ (C = 4 or 5)	Segmentation available; used optionally during training.
Validation	125	T1, T1Gd, T2, FLAIR	4 × 128^3^	4 × 64^3^	No segmentation; used for unsupervised evaluation.

**Table 2:** Demographic and clinical characteristics of the BraTS 2020 cohort. Summary of patient demographics and outcome variables derived from the unified metadata (*metadata_final.csv*). Values are aggregated by dataset split (training and validation). Mean age ± standard deviation (SD) and resection categories (GTR = gross total resection, STR = subtotal resection, NA = not available) are reported where available. Survival information was analyzed only for the training subset (see Section 2.9, Section 2.10, Section 2.11 and Section 2.12).

Split	Subjects (n)	Mean Age ± SD (Years) *	GTR (n)	STR (n)	NA (n)
Training	369	61.22 ± 11.87	119	10	0
Validation	125	57.27 ± 14.58	29	0	0
Total	494	60.79 ± 12.23	148	10	0

* Age available for 265/494 subjects.

**Table 3:** Structure of the unified embedding table.

Field	Description
split	Dataset partition (train/valid)
case_id	Unique subject identifier
npz_path_resolved	Path to the corresponding preprocessed MRI NPZ file
stability	Decoder sensitivity metric quantifying local latent manifold flatness
z0…z127	128-dimensional latent embedding features produced by the trained autoencoder
surv_days	Overall survival time (days)
event	Survival event indicator (1 = death, 0 = censored)
age	Patient age at diagnosis
resection	Extent of surgical resection (GTR, STR, or NA)

**Table 4:** Ridge-regularized Cox proportional hazards model (training n = 236; penalizer = 0.5).

Variable	HR	95% CI	*p*-Value	Interpretation
Age	1.30	1.16–1.45	5 × 10^−6^	Older age increases hazard
Stability	1.06	0.95–1.18	0.31	Non-significant
PC1	1.00	0.98–1.02	0.73	Non-significant
PC2	1.01	0.99–1.03	0.41	Non-significant
PC3	1.01	0.98–1.04	0.62	Non-significant
PC4	0.99	0.96–1.02	0.49	Non-significant

**Table 5:** Hyperparameters and rationale.

Component	Hyperparameter	Value	Rationale/Notes
Autoencoder	Latent dimension	128	Best trade-off between reconstruction fidelity and manifold smoothness; documented in Section 2.3.
	Depth	4 Conv3D blocks	Standard for 64^3^ patches; avoids overfitting while preserving capacity.
Clustering	K (clusters)	3	Optimal via silhouette, DB, CH indices. K = 2 merges low + intermediate basins; K = 5 overfragments boundaries.
Neural-ODE	Hidden dims	[256, 128] MLP	Ensures smooth latent vector field and stable ODE integration.
	k-NN (graph supervision)	8	Captures local tangent structure without oversmoothing; matches Section 2.14.
Perturbation analysis	Kernel size	7 voxels	Matches anatomical granularity of peritumoral variations; from Section 2.13.
	Perturbation magnitude ε	0.1–0.2 relative	Produces local but non-trivial sensitivity responses across latent dimensions.
Reinforcement learning (SAC)	Time horizon	60 steps	Matches RL setup (H = 60); balances exploration depth with computational cost.

**Table 6:** Age-adjusted Cox model with attractor indicators. Ridge-regularized Cox PH including age and one-hot attractor labels (reference = Attr-0). Age is the dominant covariate; attractor terms are not individually significant after FDR.

Variable	HR	95% CI	*p*-Value	Interpretation
Age	1.016	1.008–1.024	1.1 × 10^−4^	Older age increases hazard
Attr 1	1.003	0.815–1.236	0.975	Not significant
Attr 2	1.017	0.835–1.238	0.869	Not significant

## Data Availability

The raw MRI and clinical data analyzed in this study are available as part of the BraTS 2020 Challenge (https://www.kaggle.com/datasets/awsaf49/brats20-dataset-training-validation)“URL (accessed on 25 October 2025)”. Derived embeddings, metadata tables, and morphodynamic analysis outputs generated during this research are securely stored in Google Drive. These materials can be made available upon reasonable request to the corresponding author, subject to data use and confidentiality agreements. No public repository was used due to institutional data management and privacy policies.

## Supplementary Materials

The following supporting information can be downloaded at https://www.mdpi.com/article/10.3390/diagnostics16010139/s1. Supplementary Materials (S1–S5): S1: Detailed preprocessing workflow (data normalization, patch extraction, and metadata integration); S2: Voxel-level attractor sensitivity mapping (Flip/Shift map computation and reproducibility metrics); S3: Neural-ODE and control simulation details (architecture, training, and adaptive control setup); S4: Prospective biological validation and translational integration (planned radiogenomic and biopsy correlation roadmap). S5: Permutation validation of attractor geometry—statistical validation demonstrating non-randomness of attractor structure, including permutation test methodology, distribution plots, and silhouette score comparisons between empirical and null models.
