# Supplementary material for: AI-Guided Inference of Morphodynamic Attractor-like States in Glioblastoma
Source: Diagnostics (Basel). 2026 Jan 1;16(1):139. doi: 10.3390/diagnostics16010139 (PMC12785452; doi:10.3390/diagnostics16010139)
Supplement: Supplementary file 1 [file diagnostics-16-00139-s001.zip › diagnostics-4005255-supplementary.pdf]

Supplementary Materials

S1. Detailed Preprocessing Workflow

All preprocessing scripts were implemented in **Python 3.10** using the following core libraries:NiBabel 5.2; TorchIO 0.18; NumPy 1.26; Pandas 2.2; SciPy 1.13.

Processing was performed locally on a Windows 11 workstation (Intel i7-12700K, 64 GB RAM, RTX 4090) and mirrored to **Google Drive** for GPU-accelerated training on **Google Colab (A100, CUDA 12.3)**.

S1.1 Folder Organization

```
project_root/
│
├── raw_BraTS/
│ ├── BraTS2020_TrainingData/
│ │ ├── BraTS20_Training_001/
│ │ │ ├── BraTS20_Training_001_t1.nii.gz
│ │ │ ├── BraTS20_Training_001_t1ce.nii.gz
│ │ │ ├── BraTS20_Training_001_t2.nii.gz
│ │ │ ├── BraTS20_Training_001_flair.nii.gz
│ │ │ └── BraTS20_Training_001_seg.nii.gz
│ │ └── ...
│ ├── BraTS2020_ValidationData/
│ │ ├──BraTS20_Validation_001/
│ │ │ ├── BraTS20_Validation_001_t1.nii.gz
│ │ │ ├── BraTS20_Validation_001_t1ce.nii.gz
│ │ │ ├── BraTS20_Validation_001_t2.nii.gz
│ │ │ └── BraTS20_Validation_001_flair.nii.gz
│ │ └── ...
│ └── survival_info.csv, survival_evaluation.csv
│
├── preprocessed/
│ └── npz/
│ ├── train_001.npz
│ ├── train_002.npz
│ ├── ...
│ ├── valid_001.npz
│ └── valid_002.npz
│
├── metadata_final.csv
└── scripts/
├── preprocess_brats.py
└── utils_io.py
```

S1.2 Preprocessing Script Outline
Example command:

```
python preprocess_brats.py --input raw_BraTS/ \
                    --output preprocessed/npz/ \
                    --target_size 128 \
                    --patch_size 64 \
                    --normalize_percentiles 1 99
```

Key pipeline stages:

1. **Parsing and Verification:**
   Each subject directory parsed; required modalities verified (T1, T1Gd, T2, FLAIR). Missing or corrupted NIfTI headers logged and skipped.
2. **Spatial Resampling:**
   All modalities interpolated to a target grid of **128 × 128 × 128 voxels (1 mm^3 isotropic)** using trilinear interpolation. Header voxel spacing inconsistencies corrected prior to resampling.
3. **Intensity Normalization:**
   For each modality, voxel values clipped to the **1st and 99th percentiles** of non-zero voxels, then min-max scaled to **[0, 1]** independently per modality.
4. **Channel Concatenation:**
   Normalized modalities concatenated along the channel axis to produce a 4 × 128 × 128 × 128 tensor.

Optional: the BraTS segmentation mask appended as a fifth channel for certain encoder-conditioning experiments (training only).

5. **Compression:**
   Saved as NumPy .npz archives: train_XXX.npz or valid_XXX.npz

Each file contains:

- image: 4 × 128 × 128 × 128 tensor (float32)
- seg: optional 1 × 128 × 128 × 128 tensor (int16)
- subject_id: case identifier

6. **Patch Extraction:**
   During training, random **64^3** crops sampled online to augment spatial diversity and maintain GPU feasibility.

Example PyTorch snippet:

```
import torch
from torchio import RandomCrop
cropper = RandomCrop((64, 64, 64))
patch = cropper(image_tensor)  # shape (C, 64, 64, 64)
```

7. **Metadata Consolidation:**
   Clinical variables merged into *metadata_final.csv* with fields:

   subject_id, split, age, EOR_GTR, EOR_STR, EOR_NA,
   surv_days, event

S1.3 Quality Control Summary

- **Subjects parsed:** 494
- **Modality completeness:** 100 % (all four MR types present)
- **Resampling failures:** 0
- **Intensity normalization outliers:** < 1 % voxels per scan truncated
- **Final dataset:** 369 training + 125 validation subjects

S1.4Reproducibility Notes

- All random seeds (Python, NumPy, Torch) fixed at 42.
- Preprocessed data verified via voxel-wise checksum after compression.
- Processing time: ≈ 3 min/subject on local CPU (Intel i7).
- Approx. dataset size: ~ 48 GB compressed.

S1.5Example Verification Plot

A script (qc_check.ipynb) generates per-subject montage views showing T1Gd, T2, FLAIR, and segmentation overlays before and after normalization to confirm geometric alignment.

S1.6 Availability

All preprocessing scripts and configuration files will be made available in google drive upon publication.

S2. Voxel-Level Attractor Sensitivity Mapping

S2.1 Overview

The attractor saliency pipeline (Section 2.13) quantifies voxel-wise sensitivity of the trained encoder's latent embedding to localized input perturbations.

Each subject-level .npz file was processed to generate:

- **flip_heat.npy** — frequency of perturbations that changed attractor label (boundary sensitivity)
- **shift_heat.npy** — mean latent displacement per voxel (encoder sensitivity)
- **meta.json** — metadata including attractor label, margin distance, perturbation parameters, and encoder tag
- **saliency_quicklook.png** — tri-planar summary visualization

A consolidated summary (saliency_summary.csv) contains per-case label, margin, and total Flip/Shift magnitudes.

Voxel-level reproducibility was assessed via two replicate runs (different perturbation amplitudes) using identical perturbation centers.

Intraclass correlation coefficients (ICC (3,1)) were computed for both Flip and Shift maps.

S2.2 Quantitative Results

Across 30 validation cases:

- **Shift ICC = 0.903 ± 0.022**, indicating high reproducibility
- **Flip ICC = 0.000 ± 0.000**, confirming attractor stability (rare hard label transitions)

Baseline gradient-based methods (Grad-CAM, Integrated Gradients) yielded negligible Dice overlap with perturbation-based maps (≈ 0.095 ± 0.0002 vs Shift; ≈ 0 overlap vs enhancing region).

S2.3 Directory Layout

```
preprocessed/
│
├── npz/
│ ├── train_001.npz
│ └── valid_001.npz
│
├── models/
│ └── ae3d_final.pth
│
├── saliency/
│ ├── train_001/
│ │ ├── flip_heat.npy
│ │ ├── shift_heat.npy
│ │ ├── meta.json
│ │ └── saliency_quicklook.png
```

```
│ ├── valid_001/
│ │ ├── ...
│ └── saliency_summary.csv
│
└── icc_voxel_maps.csv
```

S2.4 Reproduction Command Example

```
python attractor_saliency_maps.py \
   --base preprocessed \
   --model models/ae3d_final.pth \
   --splits train valid \
   --cases 1 2 3 \
   --n_perturbs 1200
```

S2.5 Outputs

All arrays are saved in NumPy .npy format (float32, shape = 128^3).
CSV summaries (icc_voxel_maps.csv, saliency_summary.csv) include per-subject statistics used for Figure 10–11.

S2.6 Code Availability

All attractor sensitivity scripts (attractor_saliency_maps.py, gradcam_ig_encoder.py) are available in google drive.

S3. Neural ODE and Control Simulation Details

S3.1 Neural ODE Field

$f_\theta(z)$: 3-layer MLP (128→256→128, ReLU); optimizer Adam (lr=10^{-3}, 60 epochs).
Integration: Runge–Kutta 4, $\Delta t=0.05$, 200 steps.

S3.2 Control Setup

Control mapping $B \in R^{(128\times8)}$, with continuous control inputs *u(t)* bounded by $||u(t)||_\infty \leq 0.1$.
Adaptive control was implemented with an off-policy **Soft Actor–Critic (SAC)** agent in PyTorch, using 2×128-unit MLPs for actor and twin critics, 8 parallel environments, horizon H=60, discount $\gamma=0.995$, and a reward combining distance to the low-risk attractor centroid and an $\ell 2$ control penalty.

S3.3 Evaluation

Redirection rate, mean terminal distance, divergence reduction reported as mean ± SD across 10 seeds.

S4. Prospective biological validation and translational integration

Overview

Although the present framework was developed using cross-sectional MRI data, the latent attractor states are hypothesized to correspond to distinct underlying biological ecosystems within glioblastoma. The following proposed analyses outline how the imaging-derived basins may be biologically anchored in future prospective studies.

Radiogenomic correlation

Each case-level attractor label will be associated with bulk RNA-seq data (when available) from The Cancer Genome Atlas (TCGA) and institutional cohorts. Gene-set variation analysis (GSVA) will quantify enrichment of proneural, classical, and mesenchymal expression programs. Multinomial logistic models, adjusted for age, tumor volume, and scanner type, will test for attractor-specific transcriptomic signatures.

Spatial biopsy integration

Voxel-level Shift maps will be co-registered with intraoperative or image-guided biopsy samples taken from enhancing cores, rims, and peritumoral edema. Immunohistochemical markers such as Ki-67, HIF-1α/CA9, CD31, and IBA1/CD68 will be used to assess proliferative, hypoxic, vascular, and inflammatory activity, respectively. Spatial correlations will determine whether areas of high latent sensitivity coincide with biologically active tumor interfaces.

Longitudinal concordance

For patients with serial imaging, latent-space displacements (Δz) or predicted ODE flow directions will be compared to transcriptomic transitions between primary and recurrent tumors, focusing on proneural-to-mesenchymal evolution trajectories.

Control-to-drug mapping

Control vectors learned in the latent space (Section 2.15) will be aligned with pathway activation scores and LINCS drug-perturbation signatures via partial least-squares or canonical correlation analysis. This will identify potential pharmacologic analogs of control directions, suggesting candidate drugs capable of steering tumors toward lower-risk attractor basins.

Summary

These biological validation steps are conceptual and not yet implemented within the current dataset. They define a translational roadmap for linking data-driven attractor dynamics with molecular mechanisms and therapeutic modulation.

S5. Permutation Test for Attractor Geometry

To evaluate whether the observed attractor structure in the latent space could arise from random clustering rather than genuine morphodynamic organization, we performed a permutation analysis of the K = 3 static attractor model. Case-level latent embeddings were used, and cluster labels were randomized while preserving the original cluster-size distribution. For each of 500 permutations, k-means was re-fit and the silhouette score was recomputed.

The empirical silhouette score (**0.573**) exceeded all permutation-based scores (mean = **0.0023 ± 0.0026**; **p = 0.002**), indicating that the attractor geometry is highly unlikely to occur by chance. The full permutation-score distribution is shown in Figure S1.

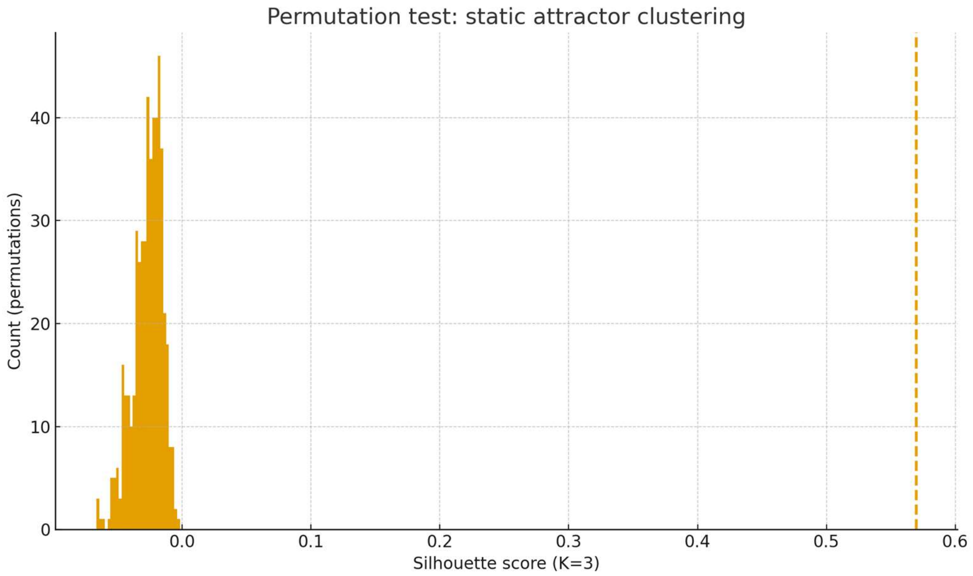

Figure S1. ***Permutation-test distribution of silhouette scores.*** Permutation test for latent attractor geometry. Histogram of silhouette scores from 500 permuted datasets in which case-level latent embeddings were randomly reassigned to cluster labels while preserving the original cluster-size distribution. The empirical silhouette score (vertical dashed line, 0.573) exceeded all permuted values (mean = 0.0023 ± 0.0026; p = 0.002), demonstrating that the attractor structure is highly unlikely to be a random artifact.
